# Cellular and Genetic Regulation of Coniferaldehyde Incorporation in Lignin of Herbaceous and Woody Plants by Quantitative Wiesner Staining

**DOI:** 10.3389/fpls.2020.00109

**Published:** 2020-03-02

**Authors:** Leonard Blaschek, Antoine Champagne, Charilaos Dimotakis, Nuoendagula, Raphaël Decou, Shojiro Hishiyama, Susanne Kratzer, Shinya Kajita, Edouard Pesquet

**Affiliations:** ^1^ Arrhenius Laboratories, Department of Ecology, Environment and Plant Sciences (DEEP), Stockholm University, Stockholm, Sweden; ^2^ Umeå Plant Science Centre (UPSC), Department of Plant Physiology, Umeå University, Umeå, Sweden; ^3^ Graduate School of Bio-Applications and Systems Engineering, Tokyo University of Agriculture and Technology, Tokyo, Japan; ^4^ Department of Forest Resource Chemistry, Forestry and Forest Products Research Institute, Tsukuba, Japan

**Keywords:** lignin, *in situ* quantification, coniferaldehyde, Wiesner test, phloroglucinol/HCl, cellular networks, image analysis

## Abstract

Lignin accumulates in the cell walls of specialized cell types to enable plants to stand upright and conduct water and minerals, withstand abiotic stresses, and defend themselves against pathogens. These functions depend on specific lignin concentrations and subunit composition in different cell types and cell wall layers. However, the mechanisms controlling the accumulation of specific lignin subunits, such as coniferaldehyde, during the development of these different cell types are still poorly understood. We herein validated the Wiesner test (phloroglucinol/HCl) for the restrictive quantitative *in situ* analysis of coniferaldehyde incorporation in lignin. Using this optimized tool, we investigated the genetic control of coniferaldehyde incorporation in the different cell types of genetically-engineered herbaceous and woody plants with modified lignin content and/or composition. Our results demonstrate that the incorporation of coniferaldehyde in lignified cells is controlled by (a) autonomous biosynthetic routes for each cell type, combined with (b) distinct cell-to-cell cooperation between specific cell types, and (c) cell wall layer-specific accumulation capacity. This process tightly regulates coniferaldehyde residue accumulation in specific cell types to adapt their property and/or function to developmental and/or environmental changes.

## Introduction

Acquired by vascular plants 450 million years ago during the colonization of land (Edwards and Axe, 2000), lignin is deposited in the different cell wall layers of specific cell types to increase their structural rigidity, resistance to degradation, and/or impermeability (Vance et al., 1980; Naseer et al., 2012; Barros et al., 2015). Lignin is believed to result from the random coupling of phenoxy radicals, formed by phenoloxidases (such as peroxidases), of predominantly **C_6_C_3_** monomers with different **C_6_** phenolic ring substitutions (hydroxyl, methoxyl, or none in 3 and 5 positions of the **C_6_** ring) and different **C_3_** aliphatic functions (acid, aldehyde, and alcohol; Boerjan et al., 2003). The specific changes in the **C_6_** and **C_3_** groups of lignin monomers are due to sequential and branching enzymatic steps grouped in a complex biosynthetic pathway (**Figure S1**). The concentration of lignin and its monomeric composition change between plant species, tissues, cell types, and different cell wall layers during development (Decou et al., 2019; Pesquet et al., 2019). In angiosperm xylem/wood, lignin concentration is high in primary cell wall layers, intermediate in secondary cell wall layers of vessels, and low in secondary cell wall layers of fibers (Terashima et al., 2012; Serk et al., 2015). Furthermore, the primary cell walls are enriched in **C_6_** phenolic unsubstituted residues (called *p*-hydroxyphenyl or **H**-units), vessel secondary cell walls in **C_6_** mono-methoxylated phenolic residues (called guaiacyl or **G**-units), and fiber secondary cell walls in **C_6_** di-methoxylated phenolic residues (called syringyl or **S**-units) (Vanholme et al., 2010). Yet, it is still undefined if the different **C_3_** aliphatic functions are as tightly spatially controlled as specific **C_6_** ring substitutions. Moreover, it remains unknown whether **C_6_C_3_** residue(s) with both specific **C_6_** ring substitution and distinct **C_3_** aliphatic function are incorporated in different ways in various cell types and cell wall layers.

Herein, we investigated the developmental and genetic regulations controlling the accumulation of coniferaldehyde, a specific **C_6_C_3_** residue with a **G** ring substitution and **C_3_** aldehyde function, at cellular and sub-cellular levels in herbaceous and woody plants. Changes in amount of coniferaldehyde residues were suggested to alter lignin biochemical, physical, and mechanical properties (Sibout et al., 2005; Holmgren et al., 2009; Fu et al., 2011; Fornalé et al., 2012; Bouvier D'Yvoire et al., 2013; Van Acker et al., 2017; Wang et al., 2018). We thus measured coniferaldehyde accumulation at the cellular levels by improving one of the oldest and most widely used histochemical methods for lignin detection, the Wiesner test (or phloroglucinol/HCl). Contrary to previous belief, we demonstrated its specificity to coniferaldehyde residues incorporated not only at the ends but also within lignin polymers, and also showed that synthetic **C_6_C_3_** monomers reacted differently than **C_6_C_3_** polymers to the Wiesner test. We hence established the quantitative capacity and set the high spatial resolution of this *in situ* method. This optimized method was used to unravel the genetic, cellular, and developmental regulation controlling the incorporation of coniferaldehyde into lignin. We thus identified for each cell type which genetic restriction(s) affected coniferaldehyde accumulation. Our findings demonstrate that coniferaldehyde incorporation into lignin during development depends on a combination of autonomous biosynthetic routes for each cell type, specific cell-to-cell cooperation between adjacent cells, and varying accumulation capacities of specific cell wall layers. Our study shows that coniferaldehyde accumulation is tightly controlled for each different lignified cell type to allow their specific cellular function(s).

## Materials and Methods

### Plant Material

*Arabidopsis thaliana* plants were grown from seeds on 1:3 (v:v) vermiculite/soil and hybrid poplar in sterile magenta^™^ boxes on 0.5 × MS medium (Duchefa, M0222.0050) with 0.4% phytagel (Sigma, P8169) in controlled growth chambers under a 16/8 h and 22°C/18°C photoperiod with 60% humidity and 150 µmol m^−2^ s^−1^ illumination. *Arabidopsis* mutants in the *Columbia-0* background included: *4cl1-1* (SALK_142526; Van Acker et al., 2013), *4cl2-4* (SALK_110197; Li et al., 2015), *ccr1-3* (SALK_123-689; Mir Derikvand et al., 2008), *cad4* (*cad-c*; SAIL_1265_A06; Lee et al., 2017), *cad5* (*cad-d*; SAIL_776_B06; Lee et al., 2017), *ccoaomt1* (SALK_151507; Kai et al., 2008), *fah1* (EMS mutant; Meyer et al., 1998), *omt1* (SALK_135290; Tohge et al., 2007), and double mutants *4cl1-1*x*4cl2-4, ccr1-3xfah1* and *cad4*x*cad5*. All *Arabidopsis* plants were genotyped to select homozygous mutants using PCR with specific primer pairs (**Table S1**). Transgenic *Populus tremula* × *tremuloides* hybrid clones T89 were transformed and micro-propagated every 3 to 4 months as previously described by Nilsson et al. (1992) with 35S driven RNA interference constructs either targeting genes *CINNAMATE-4-HYDROXYLASE* (Potri.013G157900; Bjurhager et al., 2010) or *CINNAMOYL-COA REDUCTASE* (Potri.003G181400). Characterization of silencing efficiency, developmental and biomass changes in the transgenic poplar lines used has been previously reported in Escamez et al. (2017) and the best line for each construct was selected. Two centimeters portions of stem base (8 weeks after germination for *Arabidopsis* and 4 months after micropropagation for poplar) were stored in 70% ethanol for sectioning. 2-3 cm wide branches of mature spruce trees (*Picea abies*) were harvested from the university common garden.

### Chemicals

Phenolic compounds included **H**-based compounds: **C_6_C_1_-**acid/*p-*hydroxybenzoic acid (Aldrich, H20059), **C_6_C_3_-**aldehyde/*p-*coumaraldehyde (Toronto Research Chemicals, C755450), and **C_6_C_3_-**acid/*p-*coumaric acid (Sigma, C9008); **G**-based compounds: **C_6_C_1_-**aldehyde/vanillin (Sigma-Aldrich, V1104), **C_6_C_1_-**acid/vanillic acid (Sigma-Aldrich, 94770), **C_6_C_3_-**aldehyde/coniferaldehyde (Aldrich, 382051), **C_6_C_3_-**alcohol/coniferyl alcohol (Aldrich, 223735), **C_6_C_3_-**acid/ferulic acid (Aldrich, 12870-8), and **C_6_C_3_-**ester/ethyl ferulate (Aldrich, 320617); and **S**-based compounds: **C_6_C_1_-**aldehyde/syringaldehyde (Aldrich, S7602), **C_6_C_1_-**acid/syringic acid (Sigma, S6881), **C_6_C_3_-**aldehyde/sinapaldehyde (Sigma-Aldrich, 382159), **C_6_C_3_-**alcohol/sinapyl alcohol (Aldrich, 404586), and **C_6_C_3_-**acid/sinapic acid (Aldrich, D7927). LC grade solvents were: N,N-dimethylformamide (Sigma-Aldrich, 270547), acetonitrile (EMD Millipore, 100029), methanol (EMD Millipore, 106035), and formic acid (EMD Millipore, 533002), as well as lithium chloride (Aldrich, 203637), all purchased from Sigma-Aldrich. **G C_6_C_3_**-aldehyde without a *β*-unsaturation (dihydroconiferylaldehyde) was prepared using Pd-catalyzed hydrogenation of coniferylaldehyde in moderate yield. The Wiesner reagent consisted of 1% phloroglucinol (Sigma, P3502) in 99.5% ethanol mixed with 12 M HCl (1:1, v:v).

### DHP Synthesis

Dehydrogenation polymers (DHPs) were synthesized using the *Zulauf* method by incubating 6 mM of monomer with 5 µg ml^-1^ horseradish peroxidase (Sigma-Aldrich, P8375) and 5 mM H_2_O_2_ (Sigma-Aldrich, 95299) in 10 mM sodium phosphate buffer (Sigma-Aldrich, S0876) at pH 6. The mixture was incubated at room temperature overnight under 24 rpm (rotation/min) using a Mini LabRoller H5500 (Labnet, USA). DHPs were purified by centrifugation at 13,300 *g*, washing the pellet twice in ultrapure water then resuspended in methanol.

### Spectrophotometric Analyses

Liquid spectrophotometric analyses were performed using a Hidex Sense plate reader using UV transparent 96-well plates (ThermoScientific, Sweden) for (i) monomers and DHPs before and after mixing with equal parts of Wiesner reagent, as well as (ii) for the collected LC fractions before and after adding equal parts of 6 M HCl. Solid spectrophotometric analyses were performed by drying 200 µl of DHPs onto Whatman^®^ 3MM paper and measuring in an UV-2401 PC spectrophotometer fitted with an ISR-240-A Integrating Sphere Assembly (Shimadzu, Kyoto, Japan) before and after adding 100 µl of Wiesner reagent. Hue values were calculated from the UV–vis absorption spectra by transformation into transmittance spectra, XYZ, RGB, and finally HSV values using the “colorscience” R package.

### Liquid Chromatography Techniques

For HPLC-DAD, reaction products of phenolic monomers or DHPs were injected in a Prominence LC system (Shimadzu Co., Kyoto, Japan) fitted with a Restek Raptor^™^ Biphenyl column (2.7 µm, 150 × 4.6 mm)/Restek Raptor C18 column-guard (2.7 µm, 5 × 4.6 mm) kept at 40°C, and separated with a mobile phase gradient of 0.1% formic acid in water (A) and 0.1% formic acid in methanol (B) at a flow rate of 0.6 ml min^-1^: initial condition, 20% B; to 15 min, 30% B; to 32 min, 34% B; to 40 min, 44% B; to 50 min, 50% B, to 57.5 min, 99% B, to 58.5 min, back to initial conditions for equilibration. Elution was monitored using a SPD-M20A Diode Array Detector (DAD) at 280 nm with the flow cell kept at 40°C. Peak integration analyses were made using LabSolution v5.87 (Shimadzu Co., Kyoto, Japan). Fractionation of eluting compounds was made using a FRC-10A fraction collector set at 300 µl per peak.

UPLC-MS/MS analyses were carried out using an Acquity UPLC system coupled to a Xevo TQ mass spectrometer (MS) under the control of MassLynx software (Waters Co., Milford, MA, USA). Samples were separated on an Acquity UPLC BEH C18 column (1.7 µm, 150 × 2.1 mm)/Acquity UPLC BEH C18 VanGuard precolumn (1.7 µm, 5 × 2.1 mm) kept at 40°C (Waters Co., Milford, MA, USA). A mobile phase gradient at a flow rate of 300 ml min^-1^ used (A) 0.1% formic acid in water/acetonitrile (99:1, v:v) and (B) 0.1% formic acid in acetonitrile/water (99:1, v:v): initial condition, 5% B; to 2 min, 10% B; to 30 min, 30% B; to 40 min, 50% B; to 43 min, 100% B; to 46 min, back to initial conditions for equilibration. Compound detection by MS was performed with an electrospray ionization source in negative ion mode with the following settings: capillary voltage, 2.4 kV; cone voltage, 22 V; desolvation temperature, 400°C; cone gas flow, 0 l h^-1^; desolvation gas flow, 800 l h^-1^. MS scans were first recorded between 50 and 1,000 m/z to detect peaks, which were integrated for quantification. The pseudo-molecular ions corresponding to the detected peak were selected for MS/MS fragmentation in daughter scan centroid mode using: collision gas flow, 0.15 ml min^-1^; collision energy, 20 V; cone voltage, 25 V; and scan time, 0.5 s. MS/MS spectra were compared with pure standards of phenolic monomers and unknown peaks were identified based on their MS/MS fragmentation pattern and *per se* reaction properties of the compounds.

The m/z of the different phloroglucinol-conjugates with phenolic aldehydes included: (i) H^+^-vanillyl-phloroglucinol (**Figure S2F**): 125(60), 135 (100); (ii) H^+^-syringyl-phloroglucinol (**Figure S2G**): 125(66), 165(100); (iii) coniferyl-(γ)-diphloroglucinol (**Figure S2A**): 285(100), 271(34), 161(70), 125(58); (iv) H^+^-coniferyl-(γ)-phloroglucinol (**Figure S2B**): 161(78), 125(38); and (v) diconiferyl-(γ)-diphloroglucinol (**Figure S2D**): 285(100), 446(58), 125(40).

For GPC-RID, DHPs were analyzed using a Prominence LC system (Shimadzu Co., Kyoto, Japan) on a PSS GRAM column (10 µm, 8 × 300 mm)/PSS GRAM precolumn (10 µm, 8 × 50 mm) kept at 50°C with a mobile phase made of 1% LiCl (Sigma-Aldrich, L9650) in dimethylformamide at a flow rate of 0.6 ml min^-1^. Elution was monitored using a RID-20A Refractive Index Detector with flow cell at 50°C. Determination of Mp, Mn, Mw, PDI, and percentage contribution were conducted using LabSolution v5.87 with the GPC add-on and calibrated using ReadyCal-Kit Poly(styrene) low (PSS-pskitr4l) with Mp = 266–66,000 Da (PSS Polymer Standards Service GmbH, Germany).

### Histochemical Analysis

Interfering pigments and extractives were removed by incubating cross-sections in 70% ethanol for several days before staining. Stems were embedded in 10% agarose (Sigma-Aldrich, A9539) and sectioned using a VT1000 vibratome (Leica, Sweden). Transverse cross-sections were stored in water at 4°C. Sections were mounted in water between a 1 mm thick microscopy glass-slide and a 150 µm thick glass coverslip and imaged. The cover slip was then removed and the section stained by adding 50 µl of Wiesner reagent before re-placing the cover slip. Live imaging was acquired using an Olympus BX60 brightfield microscope equipped with an Olympus UPFLN 40X objective (NA 0.75), an Olympus XC30 CCD color camera and yellow-corrected with a day light balanced filter (Olympus LBD, Japan). Irradiance, red/green/blue (RGB) adjustment, and gamma correction were kept constant for all image acquisitions.

### Image Analysis

Real-time live imaging measurements included (i) the hue from HSV images to evaluate the color, as well as (ii) the optical density or absorbance from the 8-bit images. Acquired images were analyzed using ImageJ (Schindelin et al., 2012) by (i) compilation into single image stacks, (ii) registration using the SIFT plugin, (iii) transformation into 8-bit images, and (iv) conversion to absorbance values using: absorbance = log_10_ (255/pixel value).

Fifty circular areas of 12 pixels (equivalent to a 0.7 µm diameter) for each cell type were measured before and during staining to determine the absorbance associated to the Wiesner test by subtracting unstained from stained absorbance values. Differences between stained and unstained images of the non-lignified phloem were used as additional adjustment. Hue measurements of the same circular areas were obtained by transforming registered stacks from RGB into hue/saturation/value (HSV) color-space. False-color images were obtained by (i) converting each pixel of the stained and unstained images into absorbance value, multiplying by 255 for visualization on an 8-bit scale and (ii) subtracting the unstained from the stained image, and artificially re-coloring the result. For the cell wall layer specific analyses, the aligned images were cropped to a region of interest and elastically realigned using SIFT feature extraction and the BUnwarpJ plugin. The aligned images were then transformed into absorbance and the unstained background image was subtracted from the stained image. In the resulting absorbance map, the profiles were measured in cell walls that were of equal width on both sides of the CML and aligned according to range. The values were classified into different cell wall layers, according to previous studies performed IFs in *Arabidopsis* (Goujon et al., 2003), as follows: CML, central 500 nm; S1, adjacent 500 nm; S2, 0.1–0.35 and 0.65–0.9 of the range normalized cell wall width; S3, 0.02–0.1 and 0.9–0.98 of the range normalized cell wall width. All measurements are available in the **Supplementary Data Sheet 2**.

### Cell-to-Cell Mathematical Models

A cell-to-cell mathematical modelling between adjacent cell types was computed using the differences in correlation between pairs of cell types when adjacent to each other or themselves. In each cell type, the Wiesner stain absorbance was measured in cell walls directly adjacent to other cell types, as well as cell walls adjacent to the same cell type. The absorbance was averaged over 20 points of 4 pixels in five biological replicates. The Pearson correlation coefficient *r* between two cell types when adjacent to themselves was then subtracted from *r* when one cell type was adjacent to the other. The relative effect of this cooperativity in each mutant was expressed by the ratio of average absorbance within one cell type to the average absorbance of the cell type when adjacent to another cell type. The arrows were shaded according to the average of this relative impact across genotypes.

### Pyrolysis/GC–MS

Pyrolysis/GC–MS analysis was performed according to Gerber et al. (2012) on 60 µg (± 10 µg) of freeze-dried ball-milled samples from the different plant genotypes and species using a PY-2020iD pyrolyzer equipped with an AS-1020E autosampler (Frontier Lab, Japan) connected to a 7890A/5975C GC/MS (Agilent, USA). Identification of pyrolysates was performed using combined libraries from Faix et al. (1987); Ralph and Hatfield (1991); Gerber et al. (2012), and Pesquet et al. (2013). The m/z of coniferaldehyde residues: 178 (99.9), 77 (61.0), 135 (52.2), 107 (47.7), 147 (40.0), 51 (35.0), 89 (27.6), 124 (27.3), 78 (5.6), and 177 (25.2).

### Raman Confocal Microspectroscopy

Raman spectra of interfascicular fiber, xylary fiber, and metaxylem vessel cell walls were acquired on 5 μm thick stem cross-sections, mounted in water between glass slide and coverslip, using a 100x objective (NA 0.9) using a confocal Raman microscope (Raman Touch-VIS-NIR, Nanophoton, Japan) with a 532 nm laser of 5 mW power. The linearly polarized laser light was focused on a 1 μm diameter spot of the secondary cell walls, avoiding cell corners and middle lamella. Spectra were measured using a CCD camera (Gatan Orius200D) behind a grating spectrometer (1,200 grooves mm^-1^), from 80 to 4000 cm^-1^ wavenumber bands with a spectral resolution of 1.6 cm^-1^, and analyzed using the RAMAN Viewer software (Nanophoton, Japan) with baseline correction and smoothing.

## Results

### Specific Detection of Coniferaldehyde Residues in Lignin Using the Wiesner Test

**Figure 1:**
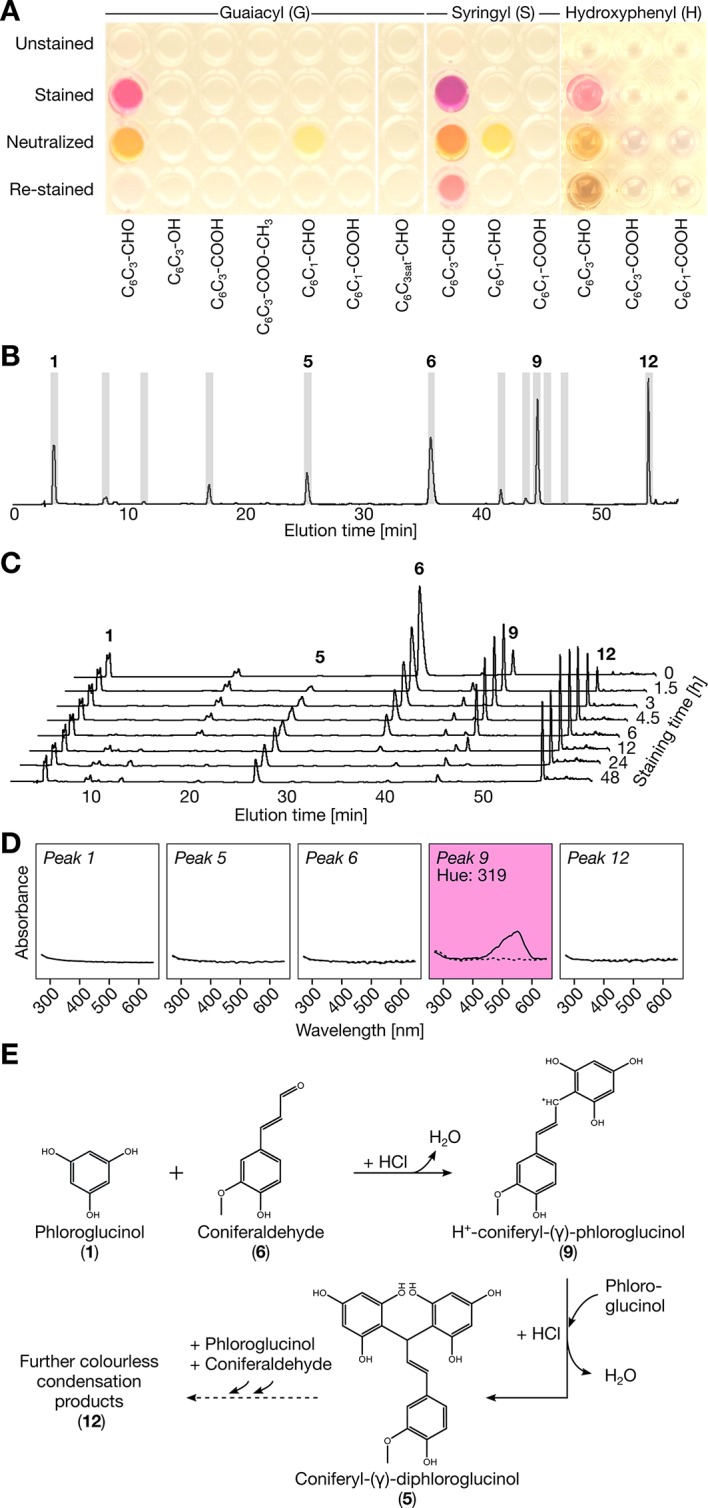
Reactivity of phenolic monomers to the Wiesner test. **(A)** Image acquired on the reactivity of 2.5 nmol of compound solubilized in methanol 1 min after adding either 6 M HCl (unstained), 0.5% phloroglucinol/6 M HCl (stained), 0.5% phloroglucinol/6 M HCl followed by 1 min neutralization with 6 M NaOH (neutralized) and followed by re-staining for 10 min in 6 M HCl (re-stained). **(B)** LC profiles of coniferaldehyde reaction to the Wiesner test in 50 mM HCl as well as the fractions collected in gray. **(C)** LC profiles showing the time-stability of coniferaldehyde reaction to the Wiesner test in 0.5% phloroglucinol/6 M HCl. The solutions were neutralized with 6 M NaOH before injection. All the chromatograms have the same absorbance scale. **(D)** Absorbance spectra of collected fractions before (dotted lines) and after addition of 6 M HCl (solid lines). Note that fraction 9 is the acid-dependent chromophore indicated by the purple background (hue = 319°). **(E)** Proposed chemical condensation reaction for the Wiesner test. Different tautomeric resonance forms of the carbocation intermediate are shown in **Figure S3E**.

Although the Wiesner test has been widely used for 140 years, its target(s) and efficiency remain uncertain as it does not reflect total lignin amount (Black et al., 1953) or aldehyde residues in lignin (Adler and Ellmer, 1948; Kim et al., 2002). However, many lignin monomers have been shown to react positively to the Wiesner test such as **G C_6_C_3_** aldehyde (coniferaldehyde), **S C_6_C_3_** aldehyde (sinapaldehyde), **H C_6_C_3_** aldehyde (*p*-coumaraldehyde), **G C_6_C_3_** without function (eugenol), and **G C_6_C_3_** alcohol (coniferyl alcohol) as well as various **G** and **S C_6_C_1_** aldehydes (Adler and Ellmer, 1948; Black et al., 1953; Ishikawa and Ide, 1954; Bland, 1966; Geiger and Fuggerer, 1979; Pomar et al., 2002; Kim et al., 2002; Varbanova et al., 2011). To solve this conundrum and determine the exact target(s) and chemical reaction behind the Wiesner test, monomer analogues were used to monitor the production of chromophore(s), their structure and stability as well as their absorbance. Monomers tested included **C_6_C_1_** and **C_6_C_3_** compounds with differences in (i) the substitution of their **C_6_** phenolic rings (**H**, **G**, or **S**), and/or (ii) in the terminal function (acid, aldehyde or alcohol) of their **C_3_** aliphatic chains. Liquid chromatography (LC) analysis before and after staining showed that compounds with **C_3_** aldehyde and alcohol but not acid could form condensation products with phloroglucinol (**Figure S2A**). However only **C_6_C_3_** aldehydes produced the typical Wiesner purple chromophore(s) at *λ*_max_ = 525 nm (hue = 343°) for **H**, *λ*_max_ = 550 nm (hue = 310°) for **G**, and *λ*_max_ = 561 nm (hue = 320°) for **S** (**Figure 1A**). The importance of the unsaturation in the **C_3_** chain was determined using dihydroconiferaldehyde, which, although condensation occurred (**Figure S2A**), did not allow the purple chromophore to form (**Figure 1A**). Analysis using LC with tandem mass spectrometry (MS/MS) showed that the purple chromophore(s) corresponded to resonance forms of H^+^-coniferyl-γ-phloroglucinol for **G C_6_C_3_** aldehyde and H^+^-sinapyl-γ-phloroglucinol for **S C_6_C_3_** aldehyde (**Figures 1B–E** and **Figure S3**). These chromophores were however unstable over time (**Figure 1C**) and their color changed with acidity (**Figure 1D** and **Figure S3**). The color fading with time was due to the formation of stable, non-chromogenic, coniferyl-γ-diphloroglucinol (**Figure 1E** and **Figure S3**). Our results thus clearly confirmed that only **C_6_C_3_** aldehyde monomers react positively to the Wiesner test.

**Figure 2:**
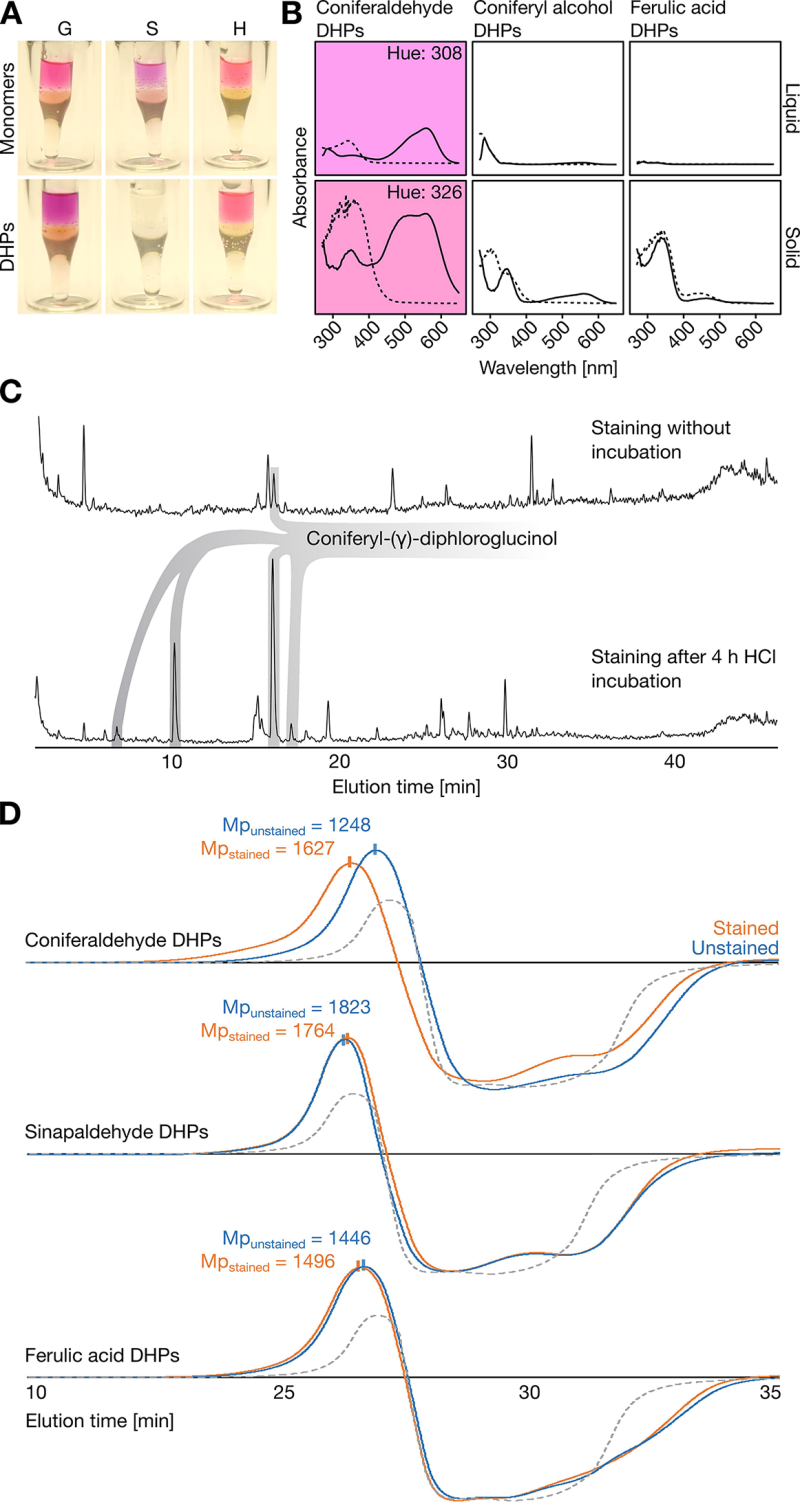
Reactivity of synthetic lignin-like dehydrogenation polymers (DHPs) with the Wiesner test. **(A)** Methanol solubilized **H**, **G**, or **S C_6_C_3_** monomers and DHPs after staining for 1 min with 0.5% phloroglucinol/6 M HCl and subsequently adding a few droplets of 6 M NaOH without mixing, thus forming a pH gradient from top to bottom. **(B)** Absorbance spectra of **G C_6_C_3_** aldehyde, alcohol, and acid DHPs solubilized in methanol (liquid) or dried onto 3MM Whatman paper (solid) and stained with the Wiesner test. Chromophores are indicated by the purple background in the corresponding hues for liquid (308°) and solid (326°) state. The small difference in hue suggests a minor influence of solvent effects on the sample reactivity to the Wiesner test. **(C)** LC-MS profiles of reaction products detected by staining with 6 M HCl/0.5% phloroglucinol following 0 and 4 h of 6 M HCl treatment. Both chromatograms are relative to total ion current. **(D)** GPC-RID profiles of **G** or **S C_6_C_3_** acid and aldehyde DHPs solubilized in methanol either unstained (un) with 6 M HCl or stained (st) with 0.5% phloroglucinol/6 M HCl (compared to unstained methanol only in gray dotted lines) before neutralization with 6 M NaOH. Mp indicates the molecular weight at the peak maximum for each condition. All chromatograms have the same intensity scale.

Since compounds generally behave very differently in monomeric and polymeric form, synthetic lignin-like DHPs of known composition were produced *in vitro* using peroxidases and either **H**, **G**, or **S C_6_C_3_** acid, aldehyde, or alcohol monomers. Unexpectedly, only **H** and **G C_6_C_3_** aldehyde DHPs reacted positively to the Wiesner test (**Figures 2A, B** and **Figure S4**). Stained **G C_6_C_3_** aldehyde DHPs produced the typical purple color of the Wiesner test in both liquid and solid states with an absorption maximum *λ*_max_ = 556–559 nm (hue = 305–330°) which faded with time (**Figure 2B**). Although **H C_6_C_3_** aldehyde is not reported as a lignin residue (Vanholme et al., 2012a), its stained DHPs had *λ*_max_ = 544 nm (hue = 330°). The color fading was investigated by LC analyses for DHPs treated by the Wiesner test with or without phloroglucinol. After a few minutes, degradation products were readily detected for all DHPs even without phloroglucinol, indicating that acidolysis occurred independently of both DHP composition and phloroglucinol (**Figure S2B**). LC–MS/MS analyses of stained **G C_6_C_3_** aldehyde DHPs with phloroglucinol revealed a gradual release of coniferyl-γ-diphloroglucinol during acidolysis (**Figure 2C**). Altogether, these results showed that the color fading of the Wiesner test over time was due to both the acidolytic break-down of lignin and the formation of stable non-chromogenic condensation products.

The fact that **S C_6_C_3_** aldehyde DHPs were unstained contradicted previous published articles claiming that both **G** and **S** aldehyde end-residues positively reacted to the Wiesner test (Pomar et al., 2002). To determine the position(s) of the residues stained, DHPs made of **G** or **S C_6_C_3_** acid or aldehyde were analyzed by gel permeation chromatography before and after staining. The molecular weight of **G C_6_C_3_** acid and **S C_6_C_3_** aldehyde DHPs were unaffected by the staining (**Figure 2D** and **Table 1**). In contrast, the molecular weight of **G C_6_C_3_** aldehyde (coniferaldehyde) DHPs exhibited a homogeneous increase after staining (**Figure 2D** and **Table 1**), which suggested that multiple residues within and at the ends of the DHP reacted with phloroglucinol. In fact, the average polymer molecular weight shift from 1,595 to 2,431 Da (**Table 1**) indicated that about 67% of its residues formed condensation products, when considering 160 Da for coniferaldehyde and 125 Da for phloroglucinol. Contrary to previous reports (Pomar et al., 2002), our results demonstrate that the Wiesner test reacts only with coniferaldehyde residues incorporated both at the ends and within lignin polymers.

**Table 1:** Polymer characterization of **C_6_C_3_ G** aldehyde, acid, and **S** aldehyde DHPs determined by gel-permeation chromatography (GPC).

	Mp (Da)		Mw (Da)		Mn (Da)		PDI	
	unst	st	unst	St	unst	st	unst	st
GCHO-DHPs	1,248	1,627	1,595	2,431	1,362	1,875	1.171	1.297
SCHO-DHPs	1,823	1,764	2,187	2,151	1,941	1,901	1.127	1.132
GCOOH-DHPs	1,446	1,496	2,329	2,326	2,096	2,082	1.111	1.117

Mp, molecular weight at the peak maximum; Mw, weight average molecular weight (Mw = Σ Ni Mi^2^ / Σ Ni Mi); Mn, number average molecular weight (Mn = Σ Ni Mi / Σ Ni); where for each elution fraction i, Mi (molecular weight) and Ni (number of molecules); PDI, polydispersity (Mw/Mn). DHPs were solubilized in methanol and allowed to react for 1 min with 6 M HCl (unstained, unst) or with 0.05% phloroglucinol/6 M HCl (stained, st), before neutralization with 6 M of NaOH and injection.

Note that compared to the Zutropf method used in Holmgren et al. (2009), Mn, Mw, and PDI of Zulauf DHPs were similar for coniferaldehyde but smaller for ferulic acid.

### *In Situ* Quantification of Incorporated Coniferaldehyde Residues in Lignin

**Figure 3:**
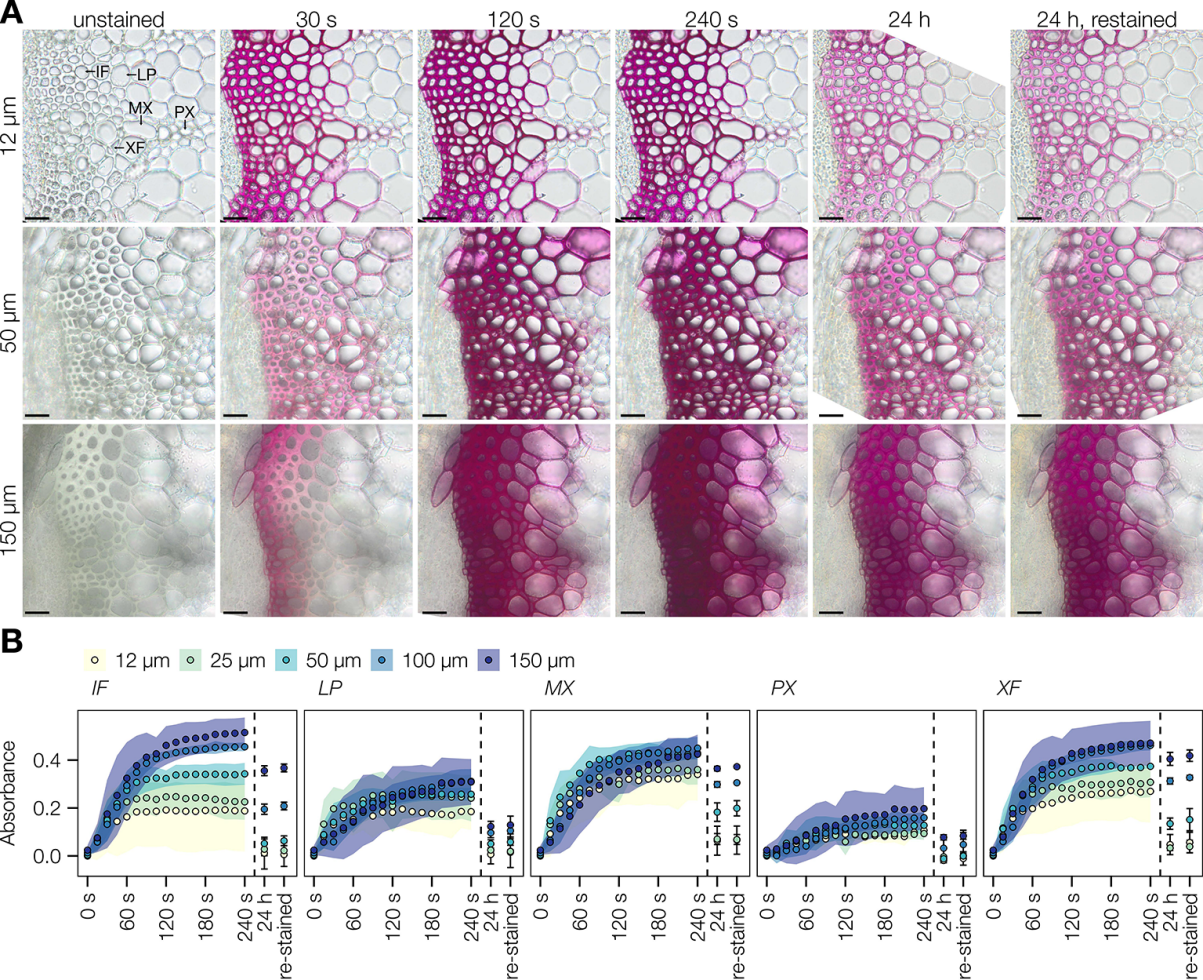
Stability of the Wiesner test in plant sections. **(A)** Live imaging of *Arabidopsis* transverse stem sections before, during, and after staining with the Wiesner test. Different section thicknesses, ranging from 12 to 150 µm, were followed for 24 h, and re-stained with addition of new Wiesner test reagent. Lignified cell types include interfascicular fibers (IF), lignified parenchyma (LP), metaxylem vessels (MX), protoxylem vessels (PX), and xylary fibers (XF). Bars = 25 µm. **(B)** Changes in absorbance in response to the Wiesner test monitored by live imaging in the five different cell types during the first 240 s after staining, after 24 h, and after re-staining. Colored ribbons and bars indicate ± standard deviation. Measurements were done using 50 measurement points of 12 pixels each per cell type in five independent plants.

**Figure 4:**
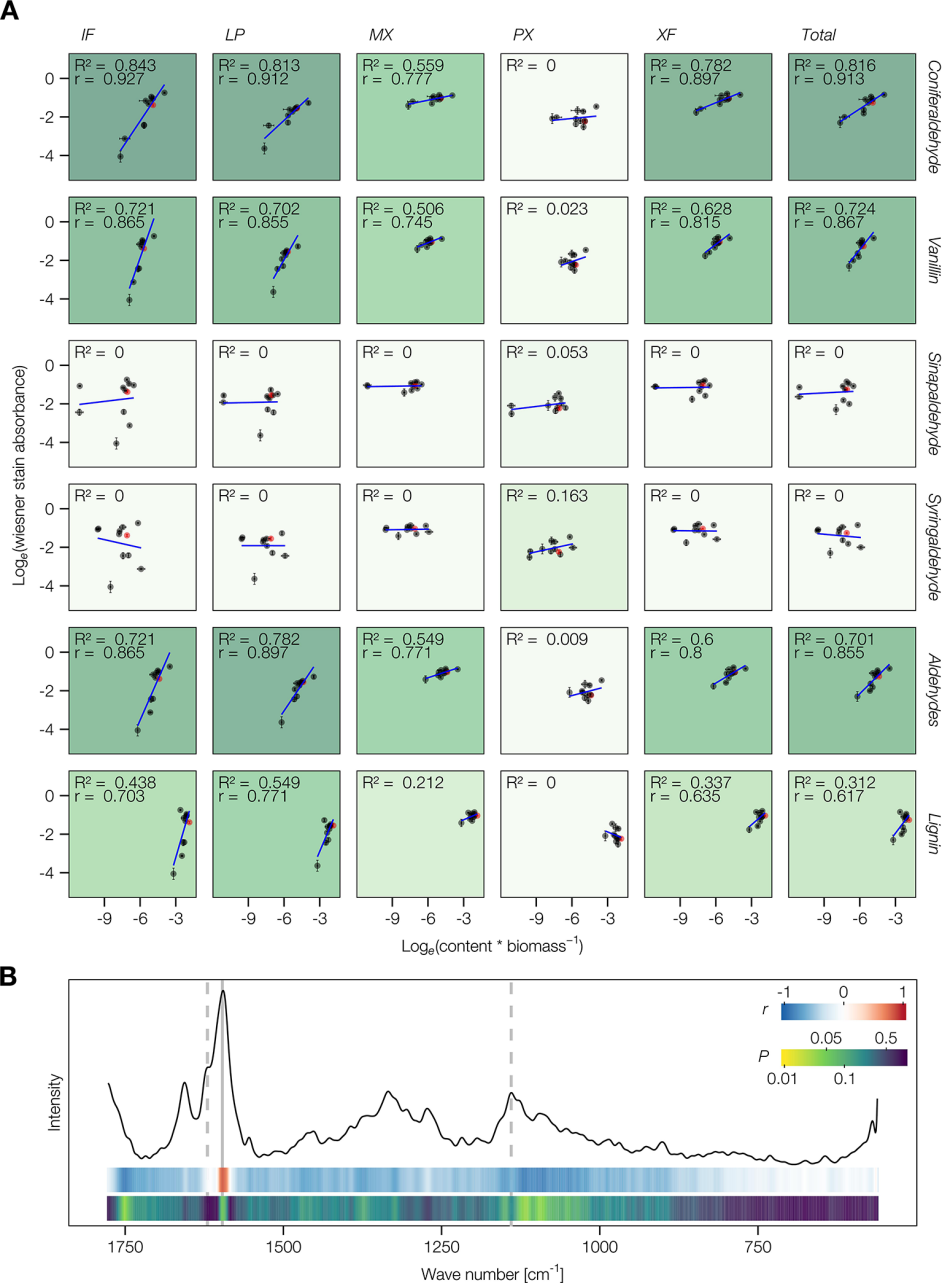
Quantitative aspect of the Wiesner test on lignin or its monomers. **(A)** Correlation analysis between the absorbance of different cell types stained by the Wiesner test and the relative content of lignin aldehydes, **G** or **S C_6_C_3_** or **C_6_C_1_** measured by pyrolysis/GC–MS. ‘Total' refers to the average across cell types weighted according to their proportional area contribution. Wild type (WT) is indicated in red. The explained variance (*R* ^2^) is indicated by the intensity of the green background and the Pearson correlation coefficient *r* is shown where its *P* < 0.05. **(B)** Comparison between the absorbance of cells stained by the Wiesner test and the microspectra obtained using Raman confocal microspectroscopy for IF, MX, and XF in WT and *4cl1*x*4cl2* mutant plants. Representative Raman microspectra of a WT metaxylem vessel cell wall is presented together with color coded ribbons showing the strength and *P*-value of the Pearson correlation between Raman intensity and Wiesner test absorbance for each wave number.

Improvement of the Wiesner test for medium-throughput *in situ* quantification of coniferaldehyde content in lignin was then evaluated on *Arabidopsis* stem cross-sections. The resolution of the method was tested in the cell walls of different lignified cell types including protoxylem vessels (PX), metaxylem vessels (MX), xylary fibers (XF), interfascicular fibers (IF), and lignified pith parenchyma (LP) (**Figure 3A**). Live-imaging of the staining in different cross-section thicknesses, ranging from 12 to 150 µm (**Figure 3A**), showed that the staining plateaued after 2 min and faded within 24 h (**Figure 3B**). Once the staining faded away, cross-sections could not be re-stained by adding new reagent (**Figure 3B**). Distribution of hue and absorbance of different stained cells across section thicknesses indicated that 50 µm thick sections presented both the smallest variation as well as the most significant differences for both parameters between cell types (**Figure S6A**). In 50 µm thick *Arabidopsis* wild-type (WT) cross-sections, absorbance per square micrometer was highest for MX and XF, ~50% less in IF and LP, and ~75% less in PX (**Figure 4B**). The produced color had a hue between 310 and 320°, similarly to coniferaldehyde as monomers and DHPs (**Figures 1A** and **2B** and **Figure S5B**). Using optimal conditions on a set of ten *Arabidopsis* loss-of-function (LOF) mutants, the quantitative capacity of the Wiesner test was compared to lignin concentration and composition measured using pyrolysis/GC–MS on the same mutants. Comparisons showed that the changes in Wiesner test absorbance directly corresponded only to changes in coniferaldehyde concentration (ranging from 1% to 25% of total lignin, **Figure S6**), but not to changes in total lignin amounts or its concentration in **S**, **H**, sinapaldehyde (**S C_6_C_3_** aldehyde), or benzaldehydes (**G**/**S C_6_C_1_** aldehydes) residues (**Figure 4A** and **Table S2**). In fact, the correlation between Wiesner test intensity and **G** lignin residues was weakened when including **H** and/or **S** residues (**Table S2**). These results showed that the Wiesner test absorbance increases linearly to allow the direct quantification of coniferaldehyde residues in lignin *in situ* across a wide range of concentrations.

To evaluate the *in situ* spatial resolution in stem cross-sections, the Wiesner test sensitivity was compared to confocal Raman microspectroscopy. Comparison between the two technologies was performed for measurements on the cell walls of three different lignified cell types (MX, XF, and IF) in two plant genotypes (WT and the LOF mutant *4cl1*x*4cl2,* which is severely altered in lignin). The highest positive and significant correlation was observed between the Wiesner test absorbances and the 1,597 cm^-1^ Raman band height, which corresponds to **C_6_** vibration of lignin residues (Gorzsás, 2017). However, no other significant correlations were observed between the Wiesner test absorbances and other Raman band heights, not even for the band shoulders 1,620 cm^-1^ or at 1,140 cm^-1^ suggested to reflect the vibrations of all phenolic aldehydes (Agarwal et al., 2011; Gorzsás, 2017) (**Figure 4B**). These results showed that the Wiesner test, in contrast to Raman microspectroscopy, specifically detects coniferaldehyde residues *in situ* with a high spatial resolution. Stained cross-sections using the Wiesner test could thus be converted in artificial color intensities to evaluate coniferaldehyde incorporation between cell types and across tissues. This conversion revealed major differences in the incorporation levels of coniferaldehyde residues for similar cell types depending on both their adjacent cells and their position within the lignified tissue (**Figure S5C**). Altogether, these results establish the Wiesner test as the current most precise *in situ* method to discriminately detect coniferaldehyde residues incorporated in the lignin of specific cell types.

### Incorporation of Coniferaldehyde Residues in Lignin of Herbaceous Plants Follows Cell Type Specific Biosynthetic Routes

The genetic control of coniferaldehyde accumulation in the lignified cell walls of the different cell types was investigated in a set of 11 *Arabidopsis* LOF mutants altered in lignin concentration and/or composition. The different LOF mutants affected specifically the lignin monomer biosynthetic pathway by modifying (i) the **C_6_** ring substitution using mutants in *CCOAOMT1*, *FAH1*, and *OMT1*; (ii) the **C_3_** terminal function (acid, aldehyde or alcohol) using mutants in *4CL1*, *4CL2*, *CCR1*, *CAD4*, and *CAD5*; as well as (iii) both with stacked mutants with LOF in both *CCR1* and *FAH1* (**Figure S1**).

**Figure 5:**
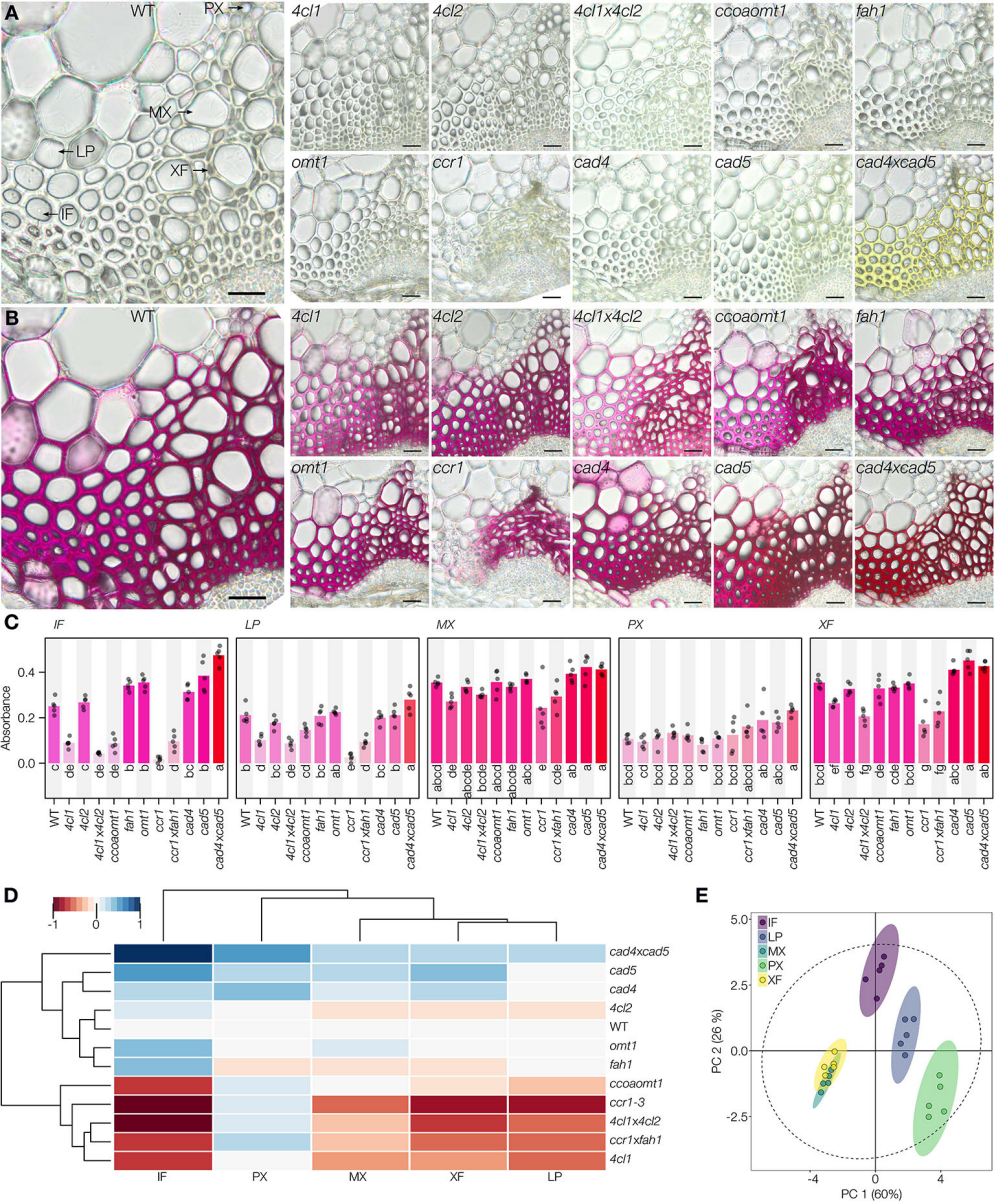
Reaction of the Wiesner test on genetically modified lignin in *Arabidopsis*. **(A, B)** 50 µm sections of the different genotypes before **(A)** and after **(B)** staining. Lignified cell types include interfascicular fibers (IF), lignified parenchyma (LP), metaxylem vessels (MX), protoxylem vessels (PX), and xylary fibers (XF). Bars = 25 µm. **(C)** Changes in absorbance in response to the Wiesner test monitored in the five different cell types for the different genotypes. Circles indicate the average of each individual replicate and bars represent the average per genotype. Quantification was done on 50 measurement points of 12 pixels each per cell type in five independent plants. The color of bars reflects the respective average color of the stain. Letters indicate significant differences according to a Tukey-HSD test (per panel; confidence limit *α* = 0.05). **(D)** Hierarchical clustering of the Wiesner test absorbance in the five lignified cell types in 12 genotypes using the range normalized Euclidean distance of their average difference from the WT. **(E)** Principal component analysis of the five lignified cell types according to their absorbance in the 12 tested genotypes; 95% data ellipses are indicated.

The analyses of absorbance per area (µm^2^) of the different stained cell types revealed that changes in **C_6_** ring substitution only significantly altered IF, reducing lignin coniferaldehyde concentration in *ccoaomt1* and increasing it in *fah1* and *omt1* compared to WT plants (**Figures 5A–C**). In contrast, the modification of **C_3_** terminal functions significantly altered the different cell types specifically for each mutation (**Figures 5A–C**). Reduced coniferaldehyde incorporation in cell wall lignin was observed in the *4cl1*, *4cl1*x*4cl2*, *ccr1*, and *ccr1*x*fah1* mutants for IF, LP, and XF, only in *ccr1* for MX but PX did not show any reduction (**Figures 5A–C**). In contrast, increased incorporation of coniferaldehyde in lignin was observed in both *cad5* and *cad4*x*cad5* mutants but not *cad4* for IF, MX, and XF, whereas PX showed increases only in *cad4*x*cad5* mutants (**Figures 5A–C**). Surprisingly, the loss of coniferaldehyde incorporation was partly reverted in *ccr1*x*fah1* for IF and LP but not in other cell types which remained like *ccr1* (**Figures 5A–C**). A striking observation was the hue in the *cad4*x*cad5* mutant both before (hue = 69°) and after (hue = 350°) staining compared to the other genotypes (**Figures S7** and **S8A**). To evaluate whether the yellow background color influenced the Wiesner test, hues of *cad4*x*cad5* mutants were analyzed using color deconvolution (Ruifrok et al., 2003) to identify the hue components before and after staining. The deconvolution of the hues merged in the stained *cad4*x*cad5* sections was made by first setting a fixed primary channel with the characteristic purple hue of the Wiesner test, which resulted in a complementary hue of 66° similar to the *cad4*x*cad5* unstained yellow background (**Figure S8B**). Inversely, setting the yellow background (hue = 64°) as a fixed primary channel resulted in a complementary hue of ~304° similar to the characteristic purple hue of the Wiesner test in WT plants (**Figure S8C**). Overall, our results revealed that the Wiesner test is not affected by sample background color and allows to unravel the impact of specific genetic mutations on the coniferaldehyde incorporation capacity of each specific cell type.

To further understand the genetic mechanisms controlling the incorporation of coniferaldehyde in the different lignified cell types, hierarchical clustering and principal component analysis (PCA) were performed to compare cell types and genotypes (**Figures 5D, E**). Surprisingly, these multivariate analyses revealed a differential influence of each mutation on the coniferaldehyde incorporation in the cell walls of each cell type (**Figures 5D, E**). Interestingly, LP, XF, and MX used similar biosynthetic routes to control the incorporation of coniferaldehyde residues in the lignin of their cell walls (**Figures 5D, E**). IF shared a similar regulation but exhibited the biggest amplitude of variation in response to LOF mutations (**Figures 5D, E**). All of these cell types reacted directly to the metabolic production and/or accumulation of coniferaldehyde. In contrast, PX presented a unique regulation of coniferaldehyde incorporation relatively unaffected by any mutation altering lignin monomer biosynthesis (**Figures 5D, E**). Altogether, our results demonstrated that different cell types possess unique biosynthetic routes to control the incorporation of coniferaldehyde residues into lignin.

### Incorporation of Coniferaldehyde Residues in Lignin of Woody Plants Changes During Tissue Development

The cell type specific incorporation of coniferaldehyde in cell walls was then investigated in woody species (hybrid poplar) genetically altered in their lignin concentration and/or composition. Constitutive RNA interference (RNAi) lines using 35S promoters were used to alter (i) the **C_6_** ring substitution by down-regulating the *C4H* gene; or (ii) the **C_3_** terminal function by down-regulating the *CCR* gene (**Figure S1**).

**Figure 6:**
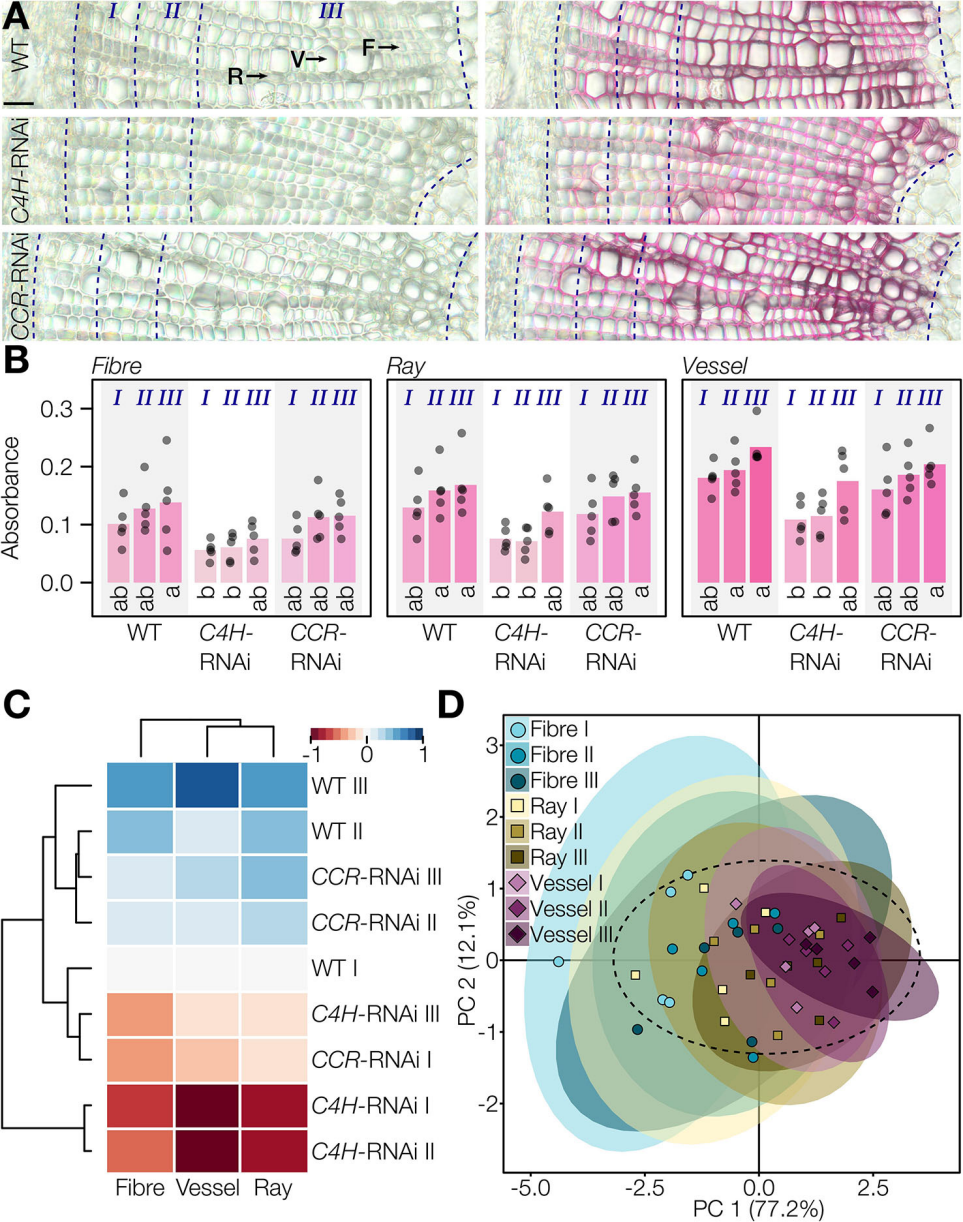
Reaction of the Wiesner test on genetically modified lignin in poplar. **(A)** Unstained and 2 min stained transverse cross-sections of poplar WT, *C4H*-RNAi, and *CCR*-RNAi plants. Rays (R), vessels (V), and fibers (F) and xylem developmental stages according to Sundell et al. (2017) are indicated by dotted lines relatively to the cambium. Bars = 25 µm. **(B)** Changes in absorbance in response to the Wiesner test monitored in the different cell types, genotypes, and along xylem development. Circles indicate the average of each individual replicate and bars represent the average per genotype. The color of bars represents the respective average color of the stain. Quantification was done on 20 measured points of 5 pixels each per cell type in five individual plants, before binning the points according to their distance to the cambium (I, 0–50 µm; II, 50–100 µm; III, 100 µm–pith). Letters indicate significant differences according to a Tukey-HSD test (per panel; *α* = 0.05). **(C)** Hierarchical clustering of Wiesner test absorbance in the three lignified cell types at the three xylem developmental stages in the three genotypes of poplar using the range normalized Euclidean distance of their average difference from the WT. **(D)** Principal component analysis of the three lignified cell types at the three xylem developmental stages according to their absorbance in the three tested genotypes; 95% data ellipses are indicated.

The Wiesner test reaction in 50 µm thick poplar stem cross-sections was similar to that in *Arabidopsis*, exhibiting the characteristic Wiesner test hue between 320 and 350° (**Figure S9**). Absorbance per area (μm^2^) between lignified cell types were measured for vessels, fibers, and rays at different distances from the cambium, in three stages marking the gradual development of xylem/wood which includes the cell death of vessels according to Sundell et al. (2017) (**Figure 6A**). In WT cross-sections, absorbance per area (µm^2^) was highest for vessels, ~25% less in rays, and ~50% less in fibers (**Figure 6B**). All cell types presented increasing absorbance per area during xylem development, thereby revealing that the cell death of vessels did not prevent the continuous incorporation of coniferaldehyde residues in their cell walls (**Figure 6B**). The lower Wiesner test absorbances measured for the poplar plants were in accordance with the fact that coniferaldehyde residues represented only 0.2 ± 0.1% of lignin residues in the WT (**Figure S6**). RNAi of lignin biosynthesis genes altered the coniferaldehyde incorporation during the development of the different cell types. All cell types exhibited significantly reduced coniferaldehyde incorporation in *C4H-*RNAi plants during the early stages of xylem development, which were then restored to the WT levels in the third developmental stage (**Figure 6B**). *CCR-*RNAi plants did not show significant changes in coniferaldehyde incorporation compared to WT plants (**Figure 6B**). Hierarchical clustering analysis and PCA were then used to evaluate the mechanisms controlling coniferaldehyde incorporation during the development of each cell type (**Figures 6C, D**). All cell types presented similar developmental regulation to control the incorporation of coniferaldehyde residues in lignin of their cell walls. However, *C4H*-RNAi had a strong influence during the entire xylem formation compared to *CCR*-RNAi which only affected the earliest stages (**Figure 6C**). Altogether, our results demonstrated that coniferaldehyde incorporation is tightly and differently regulated during the development of each lignified cell type.

### Incorporation of Coniferaldehyde Residues Into Lignin Depends on Cell Wall Layer Specific Accumulation Capacity

**Figure 7:**
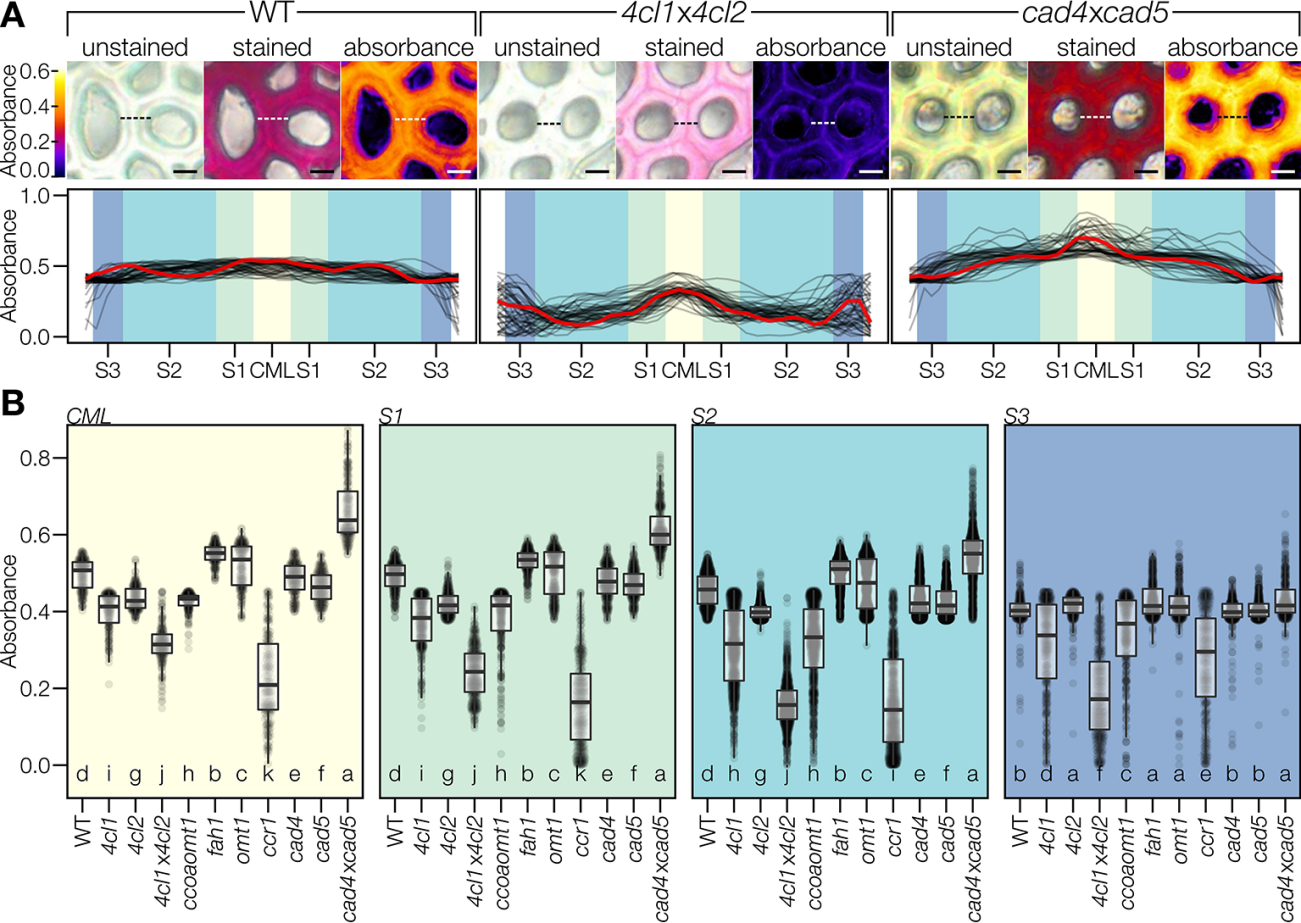
Coniferaldehyde content varies across cell wall layers. **(A)** Color coded absorbance maps created by subtracting unstained absorbance from stained absorbance. Line profiles across the cell walls separating two adjacent interfascicular fiber cells revealed the relative changes in coniferaldehyde incorporation between genotypes; such as in the coniferaldehyde-reduced mutant *4cl1*x*4cl2* and the coniferaldehyde over-accumulating mutant *cad4*x*cad5* compared to the WT. Scale bar = 5 µm. Quantification was done on 50 line profiles from two plants per genotype. The profile indicated by the dashed line in the pictures is drawn in red in the line profiles. Cell wall layers, including middle lamella (CML) and secondary cell wall layers S1–S3, are indicated by different background shading. **(B)** Coniferaldehyde incorporation in each cell wall layer. Each data point reflects one pixel in a line profile. Statistically significant differences between genotypes are indicated by different letters (per panel; Kruskal–Wallis test, Holm-adjusted for multiple comparisons, *α* = 0.05).

The spatial regulation of coniferaldehyde incorporation in lignin was then investigated by comparing the absorbances of different cell wall layers spanning across two adjacent IF. This included the primary cell wall/middle lamella (CML) in the center and three mirrored concentric secondary cell wall layers—thin S1, thick S2, and thin S3—on each side. Line profiles of absorbances were measured in the different *Arabidopsis* LOF mutants to detect spatial differences in coniferaldehyde incorporation between cell wall layers (**Figure 7A**). WT plants exhibited a homogeneous incorporation of coniferaldehyde in all cell wall layers (**Figures 7A, B**). In contrast, mutants incorporated coniferaldehyde differently in specific cell wall layers (**Figures 7A, B**). For example, *4cl1*x*4cl2* presented a reduction of ~60% in the S2 layer compared to its other layers reduced only ~30% (**Figures 7A, B**). In contrast, *cad4*x*cad5* caused a ~25% increased incorporation in CML compared to its secondary cell wall layers which were only increased ~10% (**Figures 7A, B**). Comparing all the mutants moreover indicated that the different layers did not share the same level of regulation: CML, S1, and S2 were regulated similarly but the S3 layers was less sensitive to LOF mutations affecting coniferaldehyde formation (**Figures 7A, B**). Altogether our results demonstrated that the incorporation of coniferaldehyde residues into lignin depended on the accumulation capacity of specific cell wall layers.

### Cell-to-Cell Relationships Contribute to Coniferaldehyde Incorporation in the Lignin of the Different Cell Types

**Figure 8:**
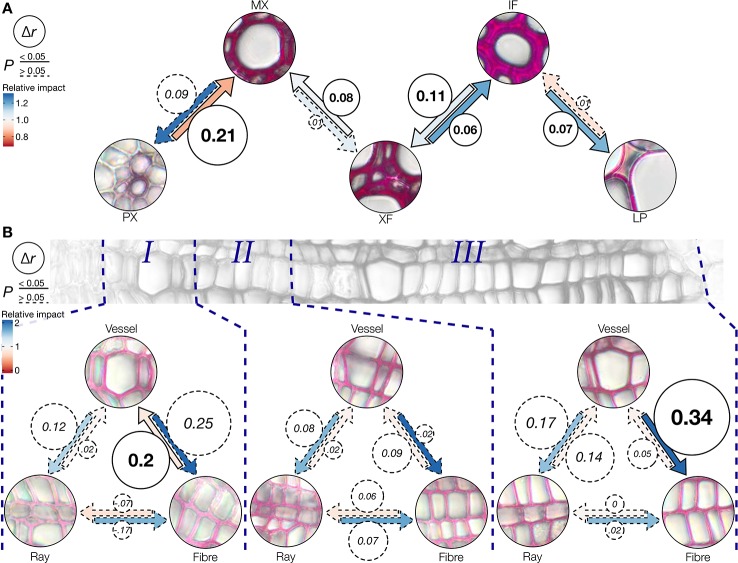
Effect of neighboring cell types on coniferaldehyde incorporation. **(A)** Schematic representation of the effect of neighboring cells on the accumulation of coniferaldehyde in specific cell types in 11 *Arabidopsis* WT and LOF lines (underlying correlations are shown in **Figure S10**). Δ*r* is shown in circles and coded in circle area and represents the changes in the Pearson correlation coefficient when the cell type at the tip of the arrow is directly adjacent to the cell type at base of the arrow. Statistically significant (William's test, *α* = 0.05) Δ*r* values are indicated by continuous arrow and circle outlines. The color of the arrows represents the relative impact (fold change in absorbance) on the cell type at the tip of the arrow when adjacent to the cell type at the base of the arrow. **(B)** Cell type-specific effects of adjacent cell types to the response to the Wiesner test in three genotypes of poplar along xylem development. Δ*r*, significance, and relative impact are coded analogous to panel **(A)**.

The differences observed in the incorporation of coniferaldehyde residues between cell types, cell wall layers, across tissues and during development after the cell death of vessels suggested the intervention of cell-to-cell cooperative processes (**Figures 5**–**7**). Since lignification depends on cell-to-cell cooperation between neighboring cell types (Pesquet et al., 2013; Serk et al., 2015), mathematical models were computed to define how specific mutation(s) altered the accumulation of coniferaldehyde residues between two neighboring cell types (**Figure 8** and **Figure S10A**). Modelling allowed to evaluate the reciprocity in the cell-to-cell cooperation between different cell type pairs as well as the contribution of the different LOF mutations on the accumulation of coniferaldehyde residues between these cell type pairs (**Figure S10B**). The cellular relationships were first investigated in *Arabidopsis* to identify the genetic restriction(s) controlling the cell-to-cell cooperation between different cell types. Coniferaldehyde incorporation in MX was affected by a unidirectional negative influence from PX and a unidirectional positive influence of XF, neither relationships highlighted any specific genetic restrictions (**Figure 8A** and **Figure S10B**). In contrast, XF and IF exhibited a reciprocal but unbalanced cooperation, essentially affected by the *ccoaomt1*, *4cl1, 4cl1*x*4cl2*, and *ccr1* mutations from XF to IF but restricted to only the *4cl1*x*4cl2* mutations from IF to XF (**Figure 8A** and **Figure S10B**). Lastly, LP also exhibited a unidirectional influence from IF mostly in *ccoaomt1* and *ccr1* but also *fah1*, *omt1*, and *cad5* mutants (**Figure 8A** and **Figure S10B**). This suggested that the accumulation of coniferaldehyde residues in LP directly depended on the metabolic availability of coniferaldehyde, further explaining the partial restoration of LP observed in the *ccr1*x*fah1* mutant (**Figures 5A–C**). The genetic regulation underlying cell-to-cell cooperation during the wood development was also investigated in poplar (**Figure 8B** and **Figure S11A**). Surprisingly, specific cell types showed an effect of neighboring cells only in certain development stages. Vessels benefited unidirectionally and positively from fibers during the early stage of xylem development (**Figure 8B** and **Figure S11B**). In contrast, fibers were influenced unidirectionally and positively from vessels only in the last stage of xylem maturation (**Figure 8B** and **Figure S11B**). Our results demonstrated the differential influence of distinct neighboring cells on the accumulation of coniferaldehyde residues during the development of other lignified cell types in both herbaceous and woody species.

## Discussion

In the present study, we demonstrate that the incorporation of coniferaldehyde residues in lignin is specifically and differently regulated between cell wall layers, cell types, across tissue and during development. These discoveries were possible because we uncovered the exact chemical reaction behind the Wiesner test 140 years after it was first described, and perfected it for *in situ* quantitative analyses with a high spatial resolution. In contrast to previous studies which concluded that both coniferaldehyde and sinapaldehyde residues at the end of polymers were stained (Pomar et al., 2002), we show that the Wiesner test reacts only with coniferaldehyde residues incorporated both at the ends and within lignin polymers. We also reveal the main reason behind this former mis-conclusion as we show that, alike the differences previously observed between amino-acid/protein, nucleotide/DNA, or glucose/cellulose, synthetic **C_6_C_3_** monomers cannot be used as standards or models for lignin polymers (**Figure 2A**).

**Figure 9:**
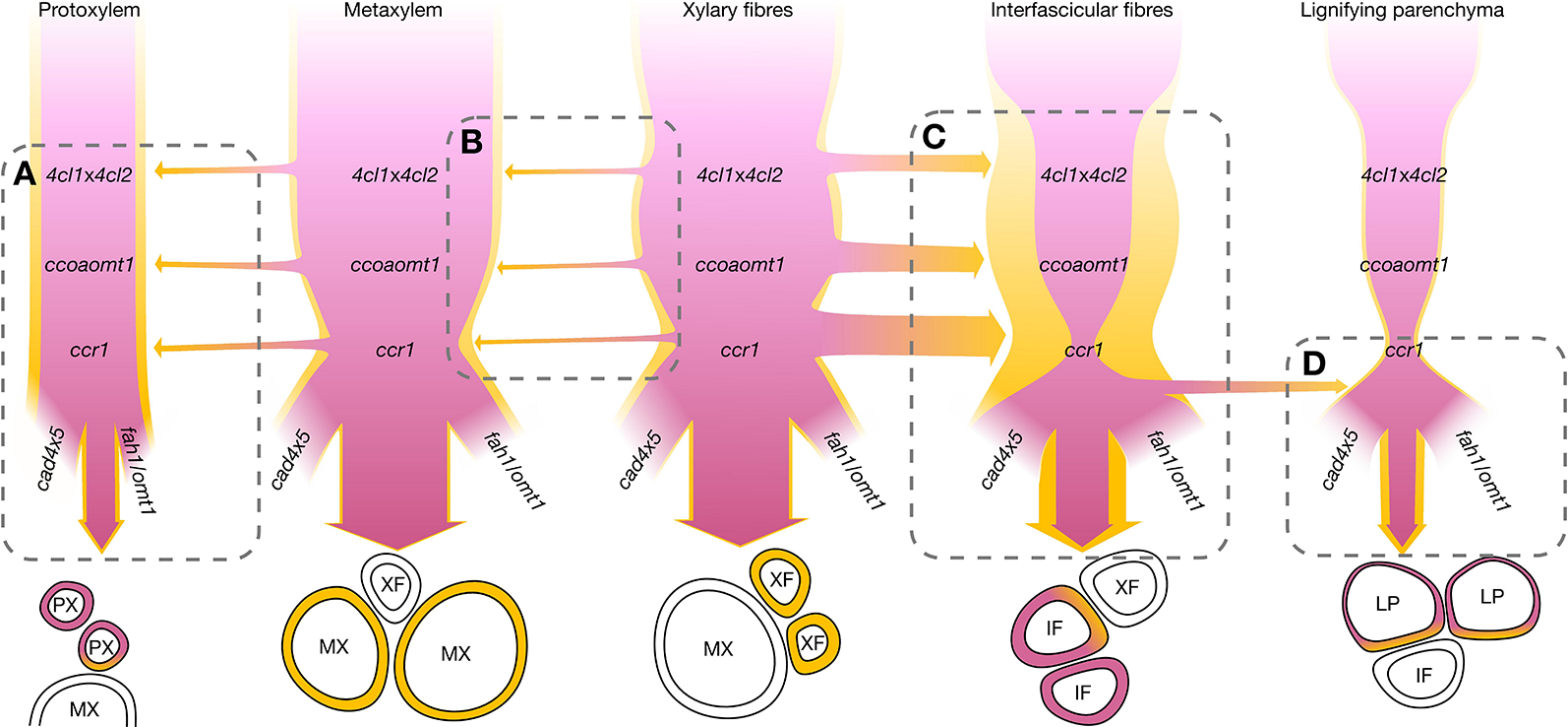
Schematic representation of the regulation of coniferaldehyde accumulation in lignin for different cell types focusing on the genetic controls of the metabolism and cooperative supply. The sequence of the mutants in the metabolic pathways leading to coniferaldehyde are shown for each cell type. The cell autonomous control of coniferaldehyde accumulation is indicated by the width of the pink ribbon modified accordingly to the effect of the different mutations. The cell cooperative control of coniferaldehyde accumulation are indicated by the width of yellow ribbon with specific neighboring cell types modified accordingly to the effect of the different mutations. Only interactions between neighboring cells are presented. **(A)** PX incorporate more coniferaldehyde when adjacent to MX independently of any particular mutations. **(B)** MX incorporates coniferaldehyde supplied by adjacent XF. The magnitude of cooperativity is largely independent of mutations, but both cell types are slightly reduced in coniferaldehyde in *ccr1.* Deciphering the relative impact of XF on MX is complicated by their constant close adjacency. **(C)** IF coniferaldehyde accumulation is highly affected by the *4cl*x*4cl2, ccoaomt1*, and *ccr1* mutations which can be bypassed by cooperation with XF. **(D)** LP behave similarly to IF without the cooperation of XF but show increased coniferaldehyde accumulation only in *cad*s, *fah1*, and *omt1* mutant plants possibly forming a metabolic sink for over-accumulating coniferaldehyde.

We also reveal that coniferaldehyde incorporation is controlled by the cell wall itself: this mechanism appears to limit the incorporation of coniferaldehyde spilling and/or diffusing across tissue to other neighboring cell types, as observed in *ccr1* mutants between XF and neighboring IF (**Figures 5**, **7**–**9**). This spatial regulation could depend on the type of phenoloxidases present in specific cell walls. Peroxidases have indeed been shown to exhibit different enzymatic affinities for **G C_6_C_3_** depending on their **C_3_** aliphatic functions (Gabaldón et al., 2005; Moural et al., 2017). We moreover show that different mutations along the biosynthetic pathway lead to different amounts of coniferaldehyde residues incorporated into specific cell wall layers (**Figure 7**). This spatial regulation could therefore also depend on the type of coniferaldehyde-containing precursor used to lignify. Metabolomic analyses of lignifying tissues in *Arabidopsis* and poplar have in fact shown the presence of many phenolic compounds containing coniferaldehyde residues such as **β-5**-linked dilignols, **β-*O-*4**/**β-5**, and **β-*O-*4**/**β-*O-*4**-linked trilignols as well as glucosides (Vanholme et al., 2012b; Sundin et al., 2014; Van de Wouwer et al., 2016; Saleme M de et al., 2017). Our results therefore question whether the same coniferaldehyde-containing precursor is used by the different cell types and cell wall layer for lignin biosynthesis.

Our study clarifies the genetic and developmental mechanisms controlling the incorporation of coniferaldehyde residues in the lignin of different cell types and species (**Figures 5**–**8**). The distinction between cell-autonomous and cell-to-cell cooperative processes is technically difficult to resolve in biological tissues. To achieve this distinction, we evaluated how distinct genetic changes altered coniferaldehyde incorporation between neighboring cell types using mathematical modelling (**Figure 8** and **Figures S10** and **11**). These models reveal that different cell-to-cell cooperation exists between different cell types and during development (**Figure 8**). Our study moreover defines the genetic restriction(s) controlling coniferaldehyde incorporation for different cooperation(s) between cell types: MX unidirectional cooperation with XF does not depend on any major genetic restriction whereas XF reciprocal cooperation with IF is greatly affected by *ccoaomt1*, *4cls* and *ccr1* mutations (**Figure S10B**). In most cases, the neighboring cells positively influence the accumulation of coniferaldehyde. However, for vessels, PX in *Arabidopsis* negatively influence coniferaldehyde accumulation in MX (**Figure 8A**) whereas, dead vessels in the most mature part of poplar xylem positively influence coniferaldehyde accumulation in fibers (**Figure 8B**). These results reveal that cooperating vessels, which are dead hollow interconnected structures conducting the sap over long distances, are able to enhance or deplete the presence of coniferaldehyde-containing lignin precursors to their neighboring cells. A simplified scheme of these complex regulations and cooperations for each specific cell type is presented in **Figure 9**. Altogether, we demonstrate that coniferaldehyde incorporation in each specific cell type depends on autonomous biosynthesis and cell-to-cell cooperation, each varying in amplitude, contribution and genetic restriction(s) depending on the cell type. Although we unraveled its genetic and cellular regulation, the molecular mechanisms (enzymes, metabolites) controlling the spatial accumulation of coniferaldehyde in specific cell wall layers still need to be clarified.

## Data Availability Statement

All datasets generated for this study are included in the article/**Supplementary Material**.

All R scripts and Fiji macros used are available at https://github.com/leonardblaschek.

## Author Contributions

EP conceived the study. LB, AC, CD, N, RD, SH, SuK, ShK, and EP performed the experiments. LB, AC, CD, ShK, and EP analyzed the data. EP wrote the article. All co-authors revised the manuscript.

## Funding

This work was supported by Vetenskapsrådet (VR) research grants 2010-4620 and 2016-04727 (to EP), the Stiftelsen för Strategisk Forskning ValueTree (to EP), and the Carl Trygger Foundation CTS 16:362/17:16/19:308 (to EP).

## Conflict of Interest

The authors declare that the research was conducted in the absence of any commercial or financial relationships that could be construed as a potential conflict of interest.

## References

[B1] Adler E. Ellmer L. (1948). Coniferaldehydgruppen im Holz und in isolierten Ligninpräparaten. Acta Chemica Scand. 2, 839–840. 10.3891/acta.chem.scand.02-0839

[B2] Agarwal U. P. McSweeny J. D. Ralph S. A. (2011). FT-Raman investigation of milled-wood lignins: softwood, hardwood, and chemically modified black spruce lignins. J. Wood Chem. Technol. 31, 324–344. 10.1080/02773813.2011.562338

[B3] Barros J. Serk H. Granlund I. Pesquet E. (2015). The cell biology of lignification in higher plants. Ann. Bot. 115, 1053–1074. 10.1093/aob/mcv046 25878140 PMC4648457

[B4] Bjurhager I. Olsson A.-M. Zhang B. Gerber L. Kumar M. Berglund L. A. (2010). Ultrastructure and mechanical properties of *Populus* wood with reduced lignin content caused by transgenic down-regulation of cinnamate 4-hydroxylase. Biomacromolecules 11, 2359–2365. 10.1021/bm100487e 20831275

[B5] Black R. A. Rosen A. A. Adams S. L. (1953). The chromatographic separation of hardwood extractive components giving color reactions with phloroglucinol. J. Am. Chem. Soc. 75, 5344–5346. 10.1021/ja01117a058

[B6] Bland D. E. (1966). Colorimetric and chemical identification of lignins in different parts of *Eucalyptus botryoides* and their relation to lignification. Holzforschung 20, 12–21. 10.1515/hfsg.1966.20.1.12

[B7] Boerjan W. Ralph J. Baucher M. (2003). Lignin biosynthesis. Annu. Rev. Plant Biol. 54, 519–546. 10.1146/annurev.arplant.54.031902.134938 14503002

[B8] Bouvier D'Yvoire M. Bouchabke-Coussa O. Voorend W. Antelme S. Cézard L. Legée F. (2013). Disrupting the *cinnamyl alcohol dehydrogenase 1* gene (*BdCAD1*) leads to altered lignification and improved saccharification in *Brachypodium distachyon* . Plant J. 73, 496–508. 10.1111/tpj.12053 23078216

[B9] Decou R. Labrousse P. Béré E. Fleurat-Lessard P. Krausz P. (2019). Structural features in tension wood and distribution of wall polymers in the G-layer of *in vitro* grown poplars. Protoplasma 257, 13–29. 10.1007/s00709-019-01416-9 31321553

[B10] Edwards D. Axe L. (2000). Novel conducting tissues in Lower Devonian plants. Bot. J. Linn. Soc. 134, 383–399. 10.1006/bojl.2000.0378

[B11] Escamez S. Latha Gandla M. Derba-Maceluch M. Lundqvist S. O. Mellerowicz E. J. Jönsson L. J. (2017). A collection of genetically engineered *Populus* trees reveals wood biomass traits that predict glucose yield from enzymatic hydrolysis. Sci. Rep. 7, 15798–15809. 10.1038/s41598-017-16013-0 29150693 PMC5693926

[B12] Faix O. Meier D. Grobe I. (1987). Studies on isolated lignins and lignins in woody materials by pyrolysis–gas chromatography–mass spectrometry and off-line pyrolysis–gas chromatography with flame ionization detection. J. Anal. Appl. Pyrolysis. 11, 403–416. 10.1016/0165-2370(87)85044-1

[B13] Fornalé S. Capellades M. Encina A. Wang K. Irar S. Lapierre C. (2012). Altered lignin biosynthesis improves cellulosic bioethanol production in transgenic maize plants down-regulated for cinnamyl alcohol dehydrogenase. Mol. Plant 5, 817–830. 10.1093/mp/ssr097 22147756

[B14] Fu C. Xiao X. Xi Y. Ge Y. Chen F. Bouton J. (2011). Downregulation of cinnamyl alcohol dehydrogenase (CAD) leads to improved saccharification efficiency in switchgrass. Bioenerg. Res. 4, 153–164. 10.1007/s12155-010-9109-z

[B15] Gabaldón C. López-Serrano M. Pedreño M. A. Barceló A. R. (2005). Cloning and molecular characterization of the basic peroxidase isoenzyme from *Zinnia elegans*, an enzyme involved in lignin biosynthesis. Plant Physiol. 139, 1138–1154. 10.1104/pp.105.069674 16258008 PMC1283753

[B16] Geiger H. Fuggerer H. (1979). Über den Chemismus der Wiesner-Reaktion auf Lignin. Z. für Naturforsch. - Sect. B. J. Chem. Sci. 34, 1471–1472. 10.1515/znb-1979-1028

[B17] Gerber L. Eliasson M. Trygg J. Moritz T. Sundberg B. (2012). Multivariate curve resolution provides a high-throughput data processing pipeline for pyrolysis–gas chromatography/mass spectrometry. J. Anal. Appl. Pyrolysis. 95, 95–100. 10.1016/j.jaap.2012.01.011

[B18] Gorzsás A. (2017). “Chemical imaging of xylem by Raman microspectroscopy,” in Methods in molecular biology. Eds. de Lucas M. Etchells J. P. (New York: Springer), 133–178. 10.1007/978-1-4939-6722-3_12 28050835

[B19] Goujon T. Ferret V. Mila I. Pollet B. Ruel K. Burlat V. (2003). Down-regulation of the AtCCR1 gene in *Arabidopsis thaliana*: effects on phenotype, lignins and cell wall degradability. Planta 217, 218–228. 10.1007/s00425-003-0987-6 12783329

[B20] Holmgren A. Norgren M. Zhang L. Henriksson G. (2009). On the role of the monolignol gamma-carbon functionality in lignin biopolymerization. Phytochemistry 70, 147–155. 10.1016/j.phytochem.2008.10.014 19056096

[B21] Ishikawa H. Ide C. (1954). Investigation on the compounds giving lignin-color reactions with phloroglucinol in the ethanol extracts of plants. Sci. Rep. Matsuyama Agric. Coll. 12, 28–32.

[B22] Kai K. Mizutani M. Kawamura N. Yamamoto R. Tamai M. Yamaguchi H. (2008). Scopoletin is biosynthesized *via ortho*-hydroxylation of feruloyl CoA by a 2-oxoglutarate-dependent dioxygenase in *Arabidopsis thaliana* . Plant J. 55, 989–999. 10.1111/j.1365-313X.2008.03568.x 18547395

[B23] Kim H. Ralph J. Lu F. Pilate G. Leplé J.-C. Pollet B. (2002). Identification of the structure and origin of thioacidolysis marker compounds for cinnamyl alcohol dehydrogenase deficiency in angiosperms. J. Biol. Chem. 277, 47412–47419. 10.1074/jbc.M208860200 12351655

[B24] Lee S. Mo H. Kim J. I. Chapple C. (2017). Genetic engineering of *Arabidopsis* to overproduce disinapoyl esters, potential lignin modification molecules. Biotechnol. Biofuels 10, 40–53. 10.1186/s13068-017-0725-0 28239412 PMC5316160

[B25] Li Y. Kim J. I. Pysh L. Chapple C. (2015). Four isoforms of *Arabidopsis* 4-coumarate:CoA ligase have overlapping yet distinct roles in phenylpropanoid metabolism. Plant Physiol. 169, 2409–2421. 10.1104/pp.15.00838 26491147 PMC4677886

[B26] Meyer K. Shirley A. M. Cusumano J. C. Bell-Lelong D. A. Chapple C. (1998). Lignin monomer composition is determined by the expression of a cytochrome P450-dependent monooxygenase in *Arabidopsis* . Proc. Natl. Acad. Sci. 95, 6619–6623. 10.1073/pnas.95.12.6619 9618461 PMC22575

[B27] Mir Derikvand M. Sierra J. B. Ruel K. Pollet B. Do C.-T. Thévenin J. (2008). Redirection of the phenylpropanoid pathway to feruloyl malate in *Arabidopsis* mutants deficient for cinnamoyl-CoA reductase 1. Planta 227, 943–956. 10.1007/s00425-007-0669-x 18046574

[B28] Moural T. W. Lewis K. M. Barnaba C. Zhu F. Palmer N. A. Sarath G. (2017). Characterization of class III peroxidases from switchgrass. Plant Physiol. 173, 417–433. 10.1104/pp.16.01426 27879392 PMC5210742

[B29] Naseer S. Lee Y. Lapierre C. Franke R. Nawrath C. Geldner N. (2012). Casparian strip diffusion barrier in *Arabidopsis* is made of a lignin polymer without suberin. Proc. Natl. Acad. Sci. 109, 10101–10106. 10.1073/pnas.1205726109 22665765 PMC3382560

[B30] Nilsson O. Aldén T. Sitbon F. Anthony Little C. H. Chalupa V. Sandberg G. (1992). Spatial pattern of cauliflower mosaic virus 35S promoter-luciferase expression in transgenic hybrid aspen trees monitored by enzymatic assay and non-destructive imaging. Transgenic Res. 1, 209–220. 10.1007/BF02524751

[B31] Pesquet E. Zhang B. Gorzsás A. Puhakainen T. Serk H. Escamez S. (2013). Non-cell-autonomous postmortem lignification of tracheary elements in *Zinnia elegans* . Plant Cell 25, 1314–1328. 10.1105/tpc.113.110593 23572543 PMC3663270

[B32] Pesquet E. Wagner A. Grabber J. H. (2019). Cell culture systems: invaluable tools to investigate lignin formation and cell wall properties. Curr. Opin. Biotechnol. 56, 215–222. 10.1016/j.copbio.2019.02.001 30849592

[B33] Pomar F. Merino F. Barceló A. R. (2002). *O*-4-linked coniferyl and sinapyl aldehydes in lignifying cell walls are the main targets of the Wiesner (phloroglucinol-HCl) reaction. Protoplasma 220, 17–28. 10.1007/s00709-002-0030-y 12417933

[B34] Ralph J. Hatfield R. D. (1991). Pyrolysis–GC–MS characterization of forage materials. J. Agric. Food Chem. 39, 1426–1437. 10.1021/jf00008a014

[B35] Ruifrok A. C. Katz R. L. Johnston D. A. (2003). Comparison of quantification of histochemical staining by hue-saturation-intensity (HSI) transformation and color-deconvolution. Appl. Immunohistochem. Mol. Morphol. 11, 85–91. 10.1097/00129039-200303000-00014 12610362

[B36] Saleme M de L. S. Cesarino I. Vargas L. Kim H. Vanholme R. Goeminne G. (2017). Silencing *CAFFEOYL SHIKIMATE ESTERASE* affects lignification and improves saccharification in *Poplar* . Plant Physiol. 175, 1040–1057. 10.1104/pp.17.00920 28878037 PMC5664470

[B37] Schindelin J. Arganda-Carreras I. Frise E. Kaynig V. Longair M. Pietzsch T. (2012). Fiji: an open-source platform for biological-image analysis. Nat. Methods 9, 676–682. 10.1038/nmeth.2019 22743772 PMC3855844

[B38] Serk H. Gorzsás A. Tuominen H. Pesquet E. (2015). Cooperative lignification of xylem tracheary elements. Plant Signaling Behav. 10, 1–5. 10.1080/15592324.2014.1003753 PMC4622721 25761224

[B39] Sibout R. Eudes A. Mouille G. Pollet B. Lapierre C. Jouanin L. (2005). *CINNAMYL ALCOHOL DEHYDROGENASE-C* and *-D* are the primary genes involved in lignin biosynthesis in the floral stem of *Arabidopsis* . Plant Cell 17, 2059–2076. 10.1105/tpc.105.030767 15937231 PMC1167552

[B40] Sundell D. Street N. R. Kumar M. Mellerowicz E. J. Kucukoglu M. Johnsson C. (2017). AspWood: high-spatial-resolution transcriptome profiles reveal uncharacterized modularity of wood formation in *Populus tremula* . Plant Cell 29, 1585–1604. 10.1105/tpc.17.00153 28655750 PMC5559752

[B41] Sundin L. Vanholme R. Geerinck J. Goeminne G. Hofer R. Kim H. (2014). Mutation of the inducible *ARABIDOPSIS THALIANA CYTOCHROME P450 REDUCTASE2* alters lignin composition and improves saccharification. Plant Physiol. 166, 1956–1971. 10.1104/pp.114.245548 25315601 PMC4256863

[B42] Terashima N. Yoshida M. Hafrén J. Fukushima K. Westermark U. (2012). Proposed supramolecular structure of lignin in softwood tracheid compound middle lamella regions. Holzforschung 66, 907–915. 10.1515/hf-2012-0021

[B43] Tohge T. Yonekura-Sakakibara K. Niida R. Watanabe-Takahashi A. Saito K. (2007). Phytochemical genomics in *Arabidopsis thaliana*: a case study for functional identification of flavonoid biosynthesis genes. Pure. Appl. Chem. 79, 811–823. 10.1351/pac200779040811

[B44] Van Acker R. Vanholme R. Storme V. Mortimer J. C. Dupree P. Boerjan W. (2013). Lignin biosynthesis perturbations affect secondary cell wall composition and saccharification yield in *Arabidopsis thaliana* . Biotechnol. Biofuels 6, 46. 10.1186/1754-6834-6-46 23622268 PMC3661393

[B45] Van Acker R. Déjardin A. Desmet S. Hoengenaert L. Vanholme R. Morreel K. (2017). Different routes for conifer- and sinapaldehyde and higher saccharification upon deficiency in the dehydrogenase CAD1. Plant Physiol. 175, 1018–1039. 10.1104/pp.17.00834 28878036 PMC5664467

[B46] Van de Wouwer D. Vanholme R. Decou R. Goeminne G. Audenaert D. Nguyen L. (2016). Chemical genetics uncovers novel inhibitors of lignification, including *p*-iodobenzoic acid targeting CINNAMATE-4-HYDROXYLASE. Plant Physiol. 172, 198–220. 10.1104/pp.16.00430 27485881 PMC5074639

[B47] Vance C. P. Kirk T. K. Sherwood R. T. (1980). Lignification as a mechanism of disease resistance. Annu. Rev. Phytopathol. 18, 259–288. 10.1146/annurev.py.18.090180.001355

[B48] Vanholme R. Demedts B. Morreel K. Ralph J. Boerjan W. (2010). Lignin biosynthesis and structure. Plant Physiol. 153, 895–905. 10.1104/pp.110.155119 20472751 PMC2899938

[B49] Vanholme R. Morreel K. Darrah C. Oyarce P. Grabber J. H. Ralph J. (2012a). Metabolic engineering of novel lignin in biomass crops. New Phytol. 196, 978–1000. 10.1111/j.1469-8137.2012.04337.x 23035778

[B50] Vanholme R. Storme V. Vanholme B. Sundin L. Christensen J. H. Goeminne G. (2012b). A systems biology view of responses to lignin biosynthesis perturbations in *Arabidopsis* . Plant Cell 24, 3506–3529. 10.1105/tpc.112.102574 23012438 PMC3480285

[B51] Varbanova M. Porter K. Lu F. Ralph J. Hammerschmidt R. Jones A. D. (2011). Molecular and biochemical basis for stress-induced accumulation of free and bound *p*-coumaraldehyde in cucumber. Plant Physiol. 157, 1056–1066. 10.1104/pp.111.184358 21940999 PMC3252134

[B52] Wang J. P. Matthews M. L. Williams C. M. Shi R. Yang C. Tunlaya-Anukit S. (2018). Improving wood properties for wood utilization through multi-omics integration in lignin biosynthesis. Nat. Commun. 9, 1579. 10.1038/s41467-018-03863-z 29679008 PMC5910405

