# Supplementary material for: Cellular and Genetic Regulation of Coniferaldehyde Incorporation in Lignin of Herbaceous and Woody Plants by Quantitative Wiesner Staining
Source: Front Plant Sci. 2020 Mar 2;11:109. doi: 10.3389/fpls.2020.00109 (PMC7061857; doi:10.3389/fpls.2020.00109)
Supplement: Supplementary file 1 [file DataSheet_1.pdf]

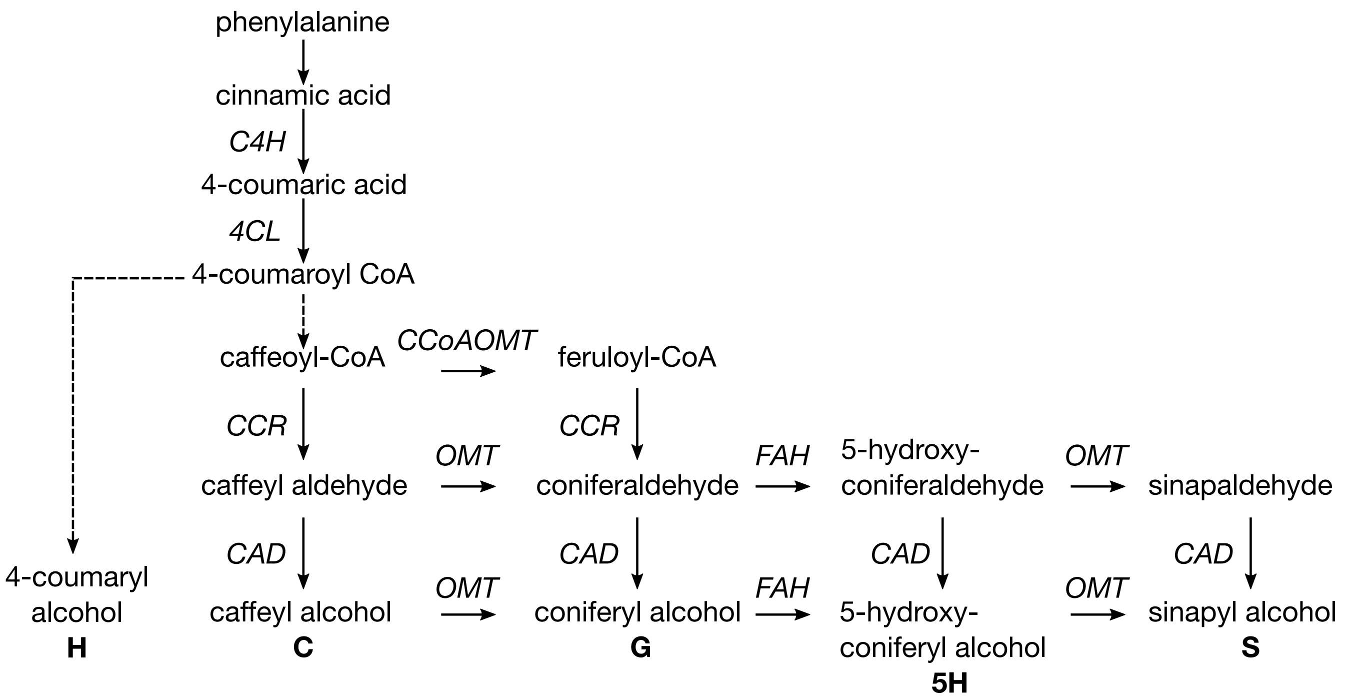

Figure S1 | Simplified scheme of the phenylpropanoid biosynthetic pathway leading to the lignin monomers. Dashed lines indicate intermediate steps for which the successive enzymes are not detailed. See Barros et al. (2015) for the complete pathway.

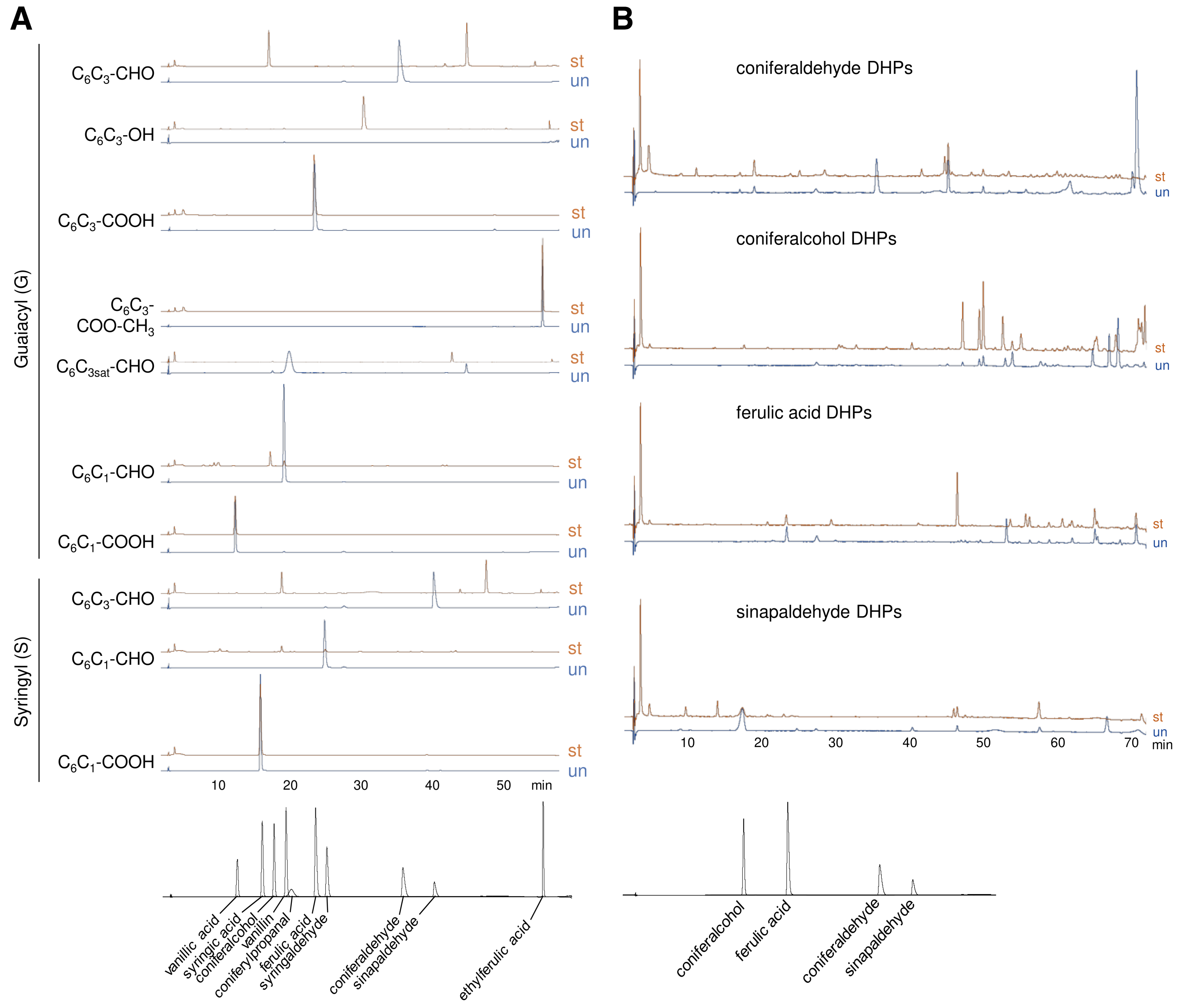

Figure S2 | Chromatographic profiles using reverse-phase chromatography of methanol-solubilised compounds neutralised with 6 M NaOH after 1 min of reaction with 6 M HCl only (un) or with 0.5 % phloroglucinol / 6 M HCl (st). Elution was monitored using a diode array detector at 280 nm.

A Monolignols and other soluble phenolic compounds.

B G C_6C_3 acid, aldehyde, alcohol and **S C_6C_3** aldehyde DHPs.

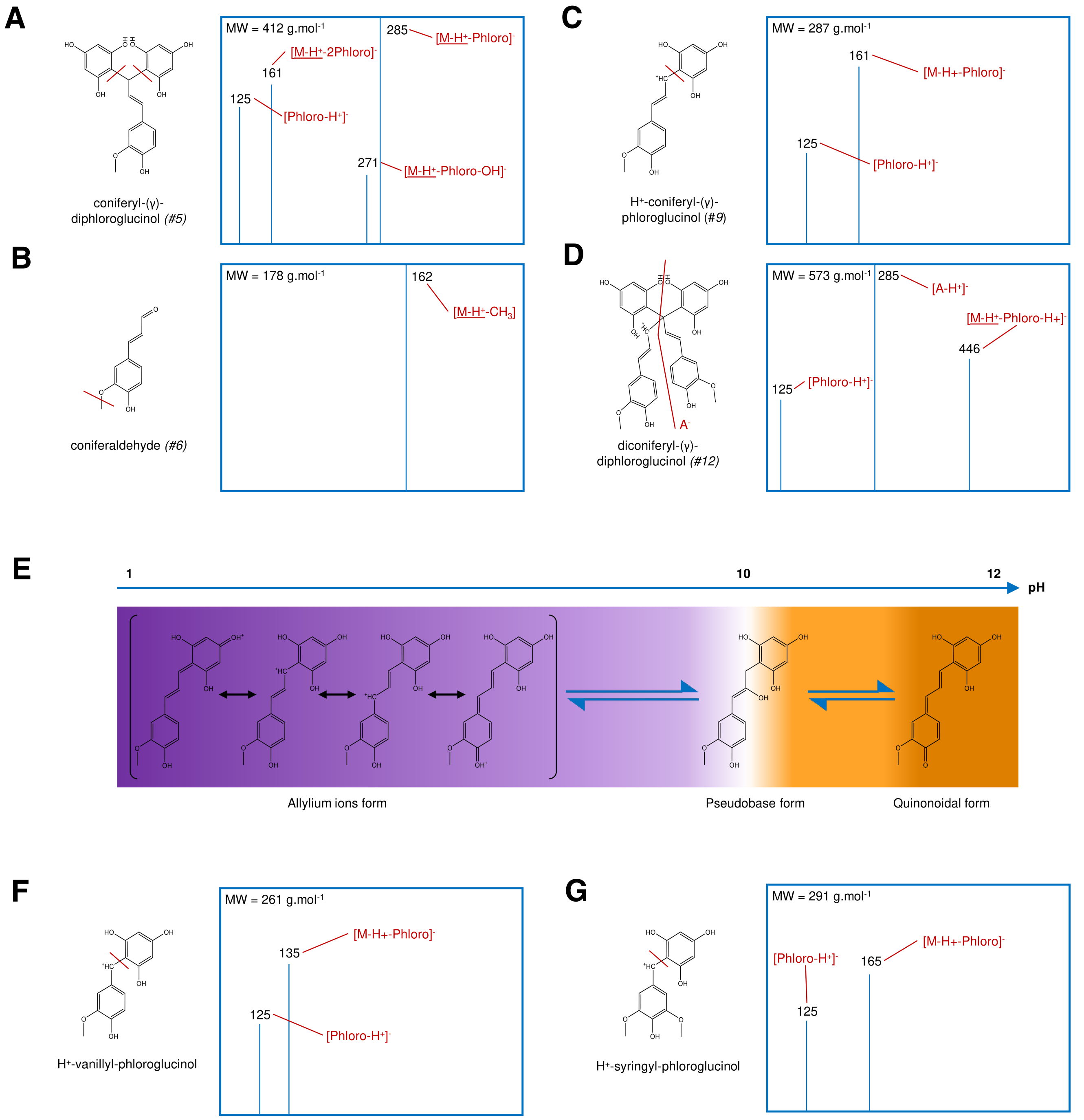

Figure S3 | Identification using MS/MS spectra of the different compounds collected in Fig. 1D from the reaction of coniferaldehyde with the Wiesner reagent in weak acid conditions.

A coniferyl-(γ)-diphloroglucinol (5)

B coniferaldehyde (6)

C H$^+$-coniferyl-(γ)-phloroglucinol (9)

D putative diconiferyl-(γ)-diphloroglucinol (12)

E Potential resonance forms of the Wiesner chromophore with coniferaldehyde (not all resonance forms shown).

F and **G** MS/MS of the benzaldehyde reaction with the Wiesner test including for vanillin: H$^+$-vanillyl-phloroglucinol (**F**), and for syringaldehyde: H$^+$-syringyl-phloroglucinol (**G**).

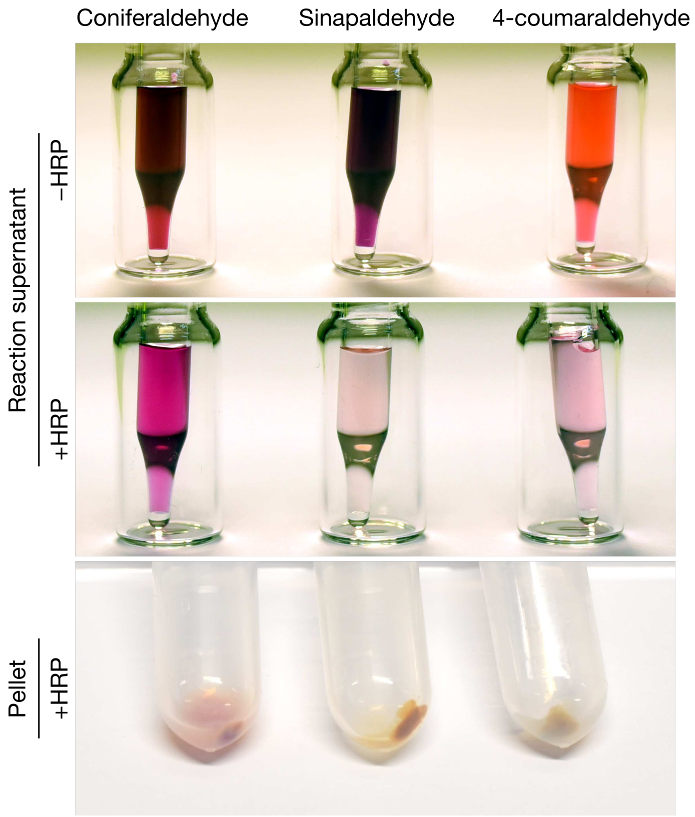

Figure S4 | Polymerisation abolishes the reaction of **S C_6C_3** aldehyde to the Wiesner test. Reactivity to the Wiesner test was evaluated on the supernatant of **C_6C_3** aldehyde DHP reaction mixtures with and without horseradish peroxidase (HRP).

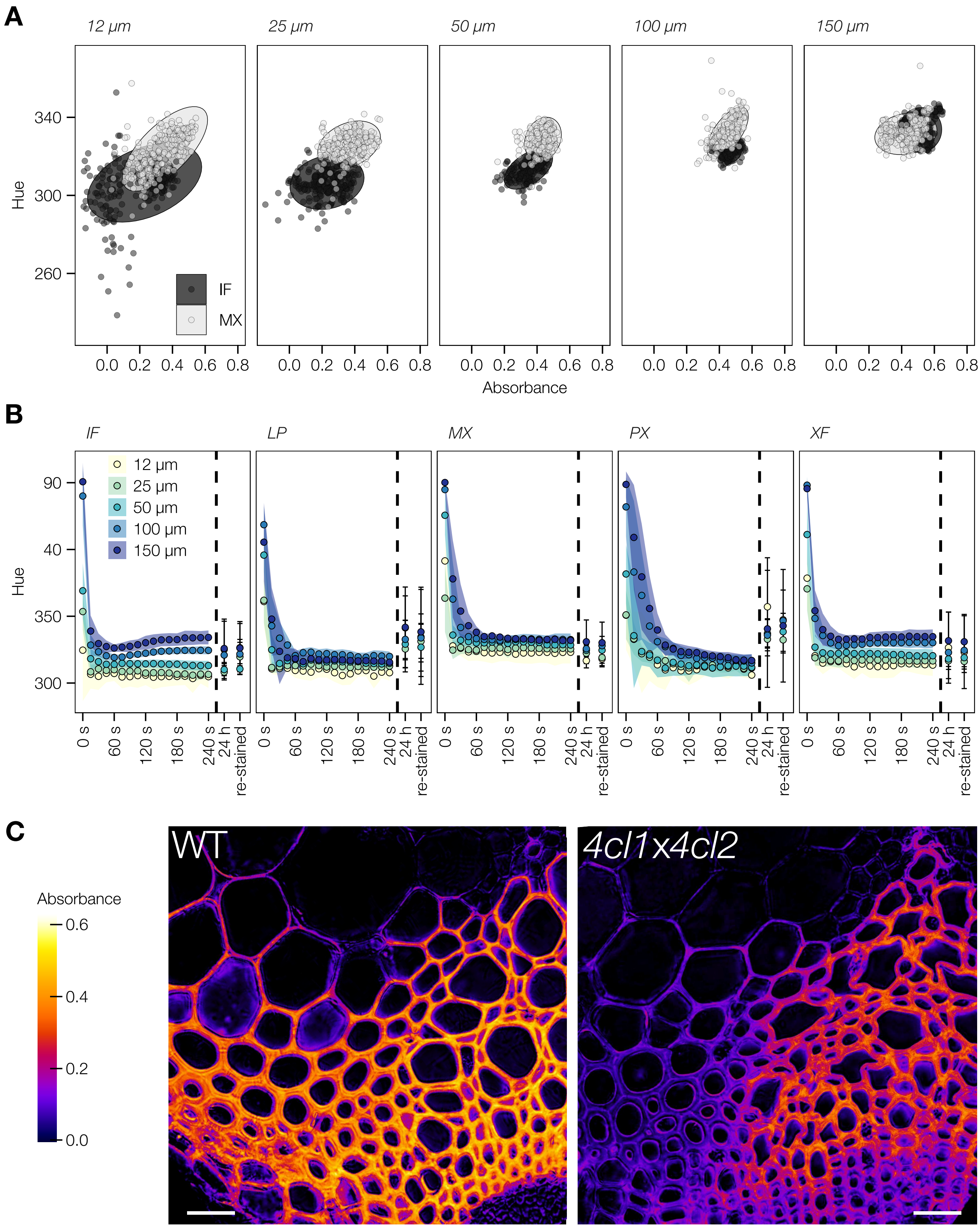

Figure S5 | Effect of section thickness on the spatial resolution and color of the Wiesner test.
A Hue plotted against absorbance for *Arabidopsis* wild type stem cross-sections. Note that 50 µm sections have less variation than thinner sections and allow to clearly distinguish between cell types.
B Live imaging of changes in the hue during staining, fading and re-staining of transverse sections of different thickness.
C Artificial color representation of coniferaldehyde content between cell types in WT and *4cl1x4cl2*.

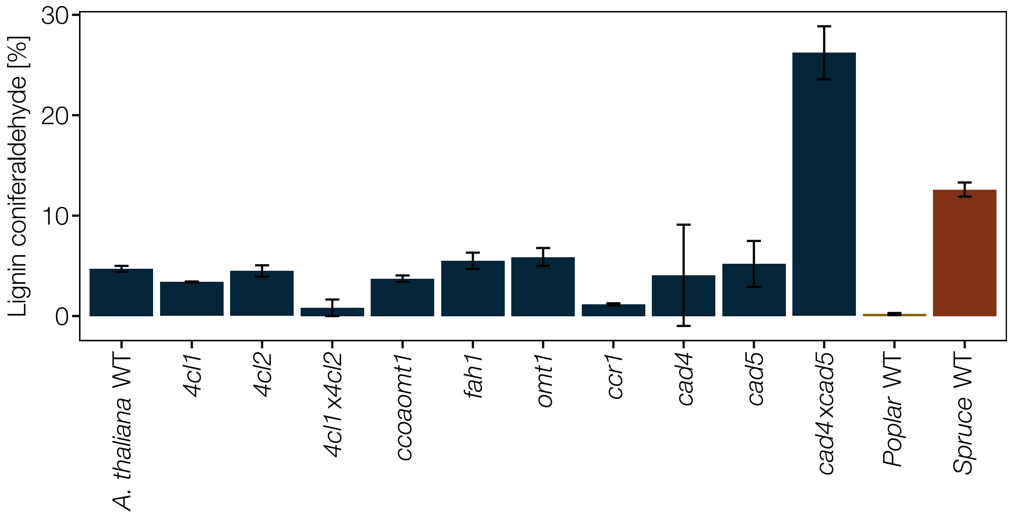

Figure S6 | Average proportion of coniferaldehyde residues in lignin according to pyrolysis-GC/MS (expressed as the ratio of coniferaldehyde specific pyrolysate peak area to total lignin pyrolysate peak area) in the different Arabidopsis LOF mutants, *in vitro Populus* stems and field grown spruce branches. Bars indicate standard deviation of $n = 3$ biological replicates.

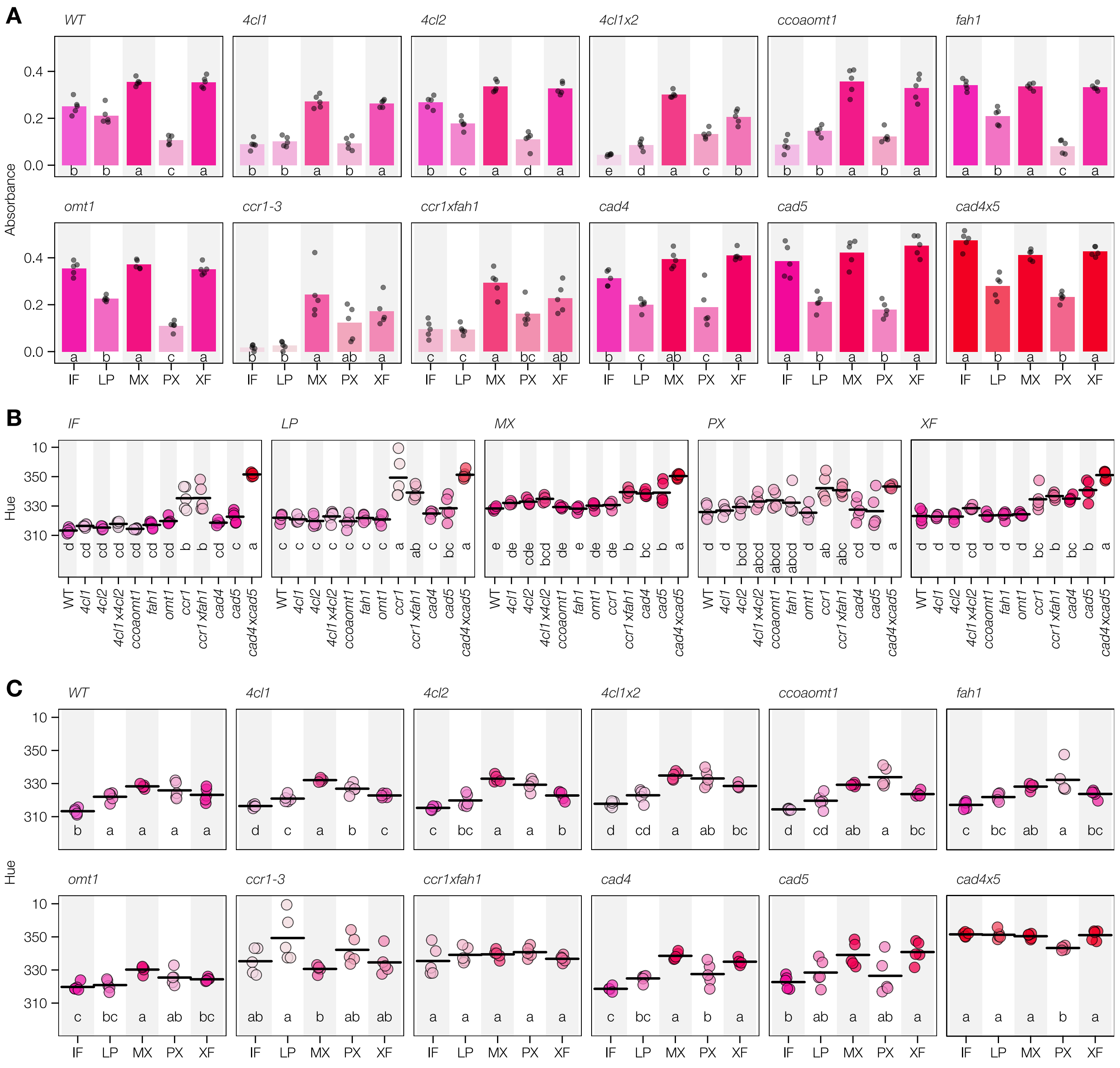

Figure S7 | Absorbance and hue of the Wiesner stain in lignin biosynthesis mutants of *A. thaliana*.
A Absorbance in the different mutants. These are the same data shown in figure 5C, but compared between the cell types within each genotype.
B Hue in the different genotypes. Circles represent averages per individual plant, the vertical bars averages per genotype.
C Hue in the different genotypes. These are the same data shown in figure S6B, but compared between the cell types within each genotype.
Letters indicate statistically significant differences between groups (per panel; Tukey-HSD test, $\alpha = 0.05$).

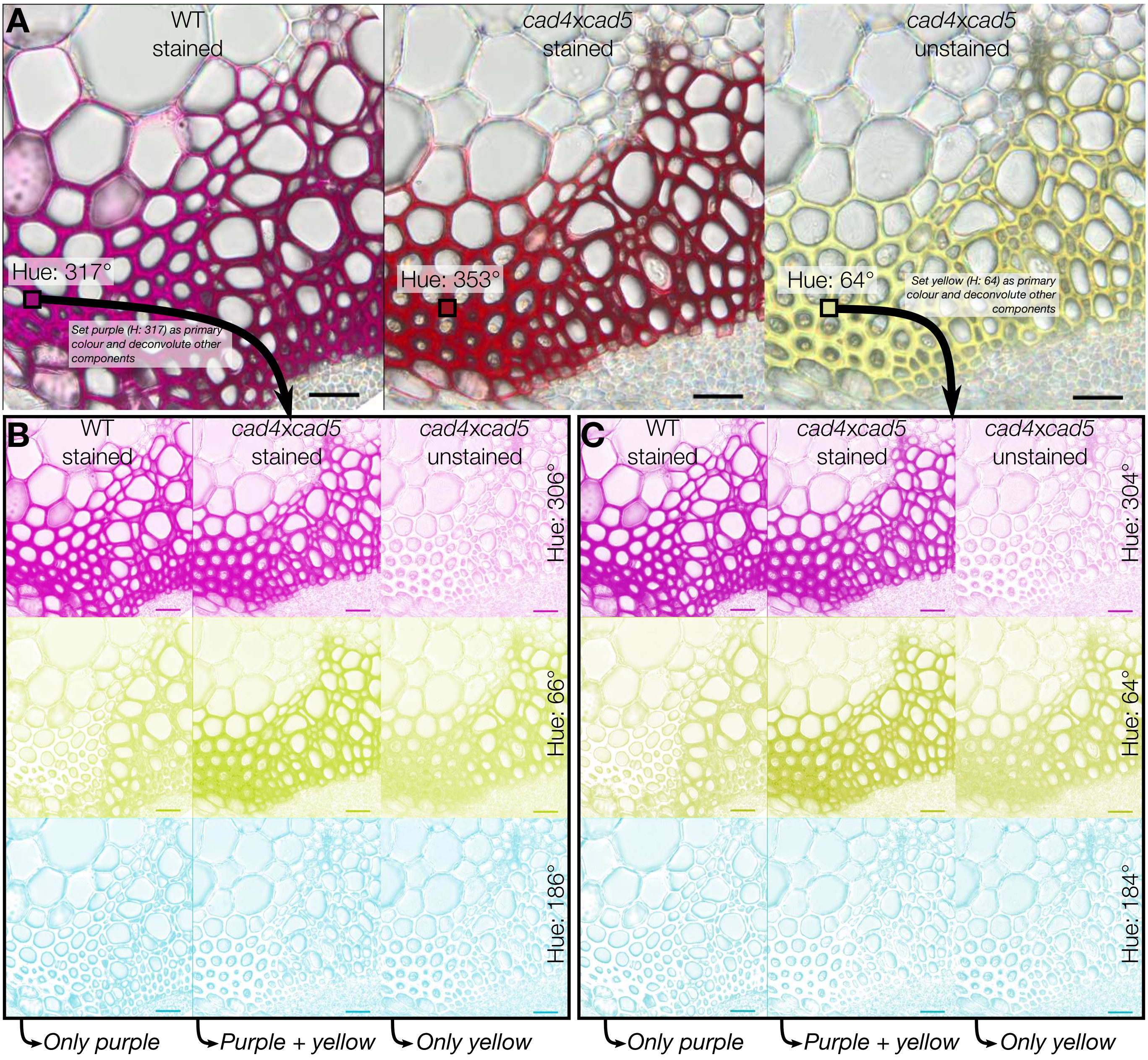

Figure S8 | Influence of background color of *cad4*x*cad5* mutant plants on the Wiesner test.

A Wiesner test stained cross-section of the WT and stained and unstained cross-sections of *cad4*x*cad5* mutant plants. The averaged hues inside the framed squares in the IF are indicated. Bars = 25 µm.

B Color deconvolution of the collage in A using the purple of the Wiesner test in the WT (hue: 317°) as primary color channel which showed that unstained *cad4*x*cad5* consisted only of yellow, stained *cad4*x*cad5* presented *cad4*x*cad5* background yellow combined with WT purple (hue: 306°) similar to the stained WT as well as a third complementary color channel (hue: 184°) which was empty in the cell wall surface of all genotypes and staining conditions.

C Color deconvolution of the collage in A using the unstained *cad4*x*cad5* (hue: 64°) as primary color channel which showed that the stained WT consisted only of purple, stained *cad4*x*cad5* presented WT purple combined with a yellow background (hue: 64°) similar to the unstained *cad4*x*cad5* as well as a third complementary color channel (hue: 184°) which was empty in the cell wall surface of all genotypes and staining conditions.

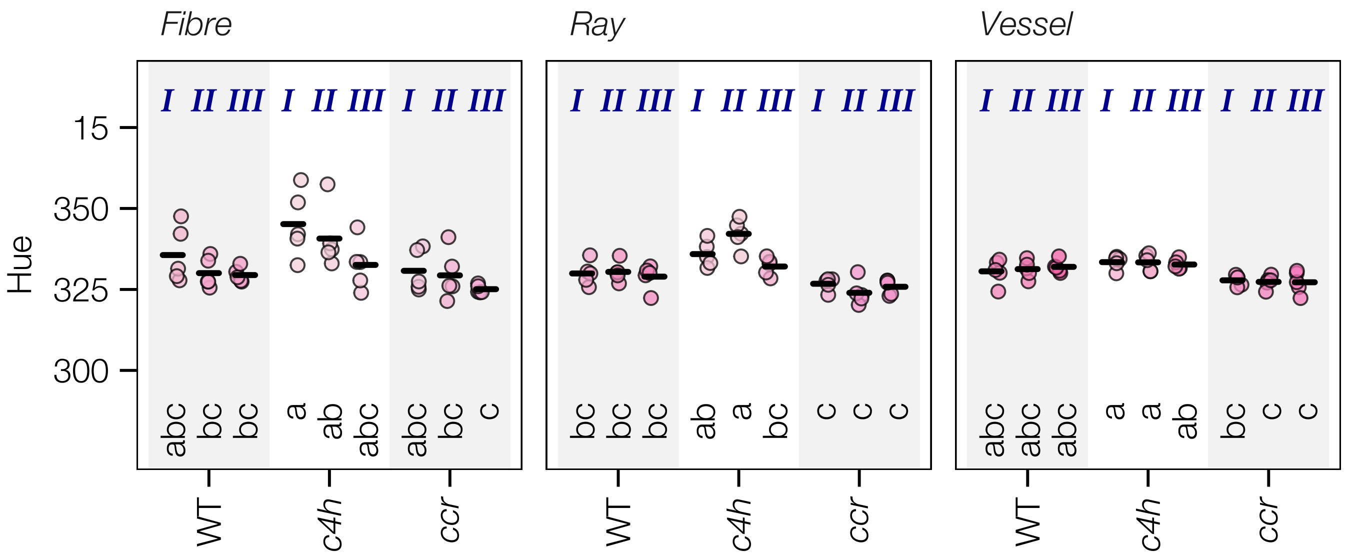

Figure S9 | Hue of the Wiesner stain in the different developmental sectors (I–III) of *P. tremuloides* × *P. tremula*. Circles represent averages per individual plant, the vertical bars averages per genotype and sector. Letters indicate significant differences according to a Tukey-HSD test (per panel; $\alpha = 0.05$).

A

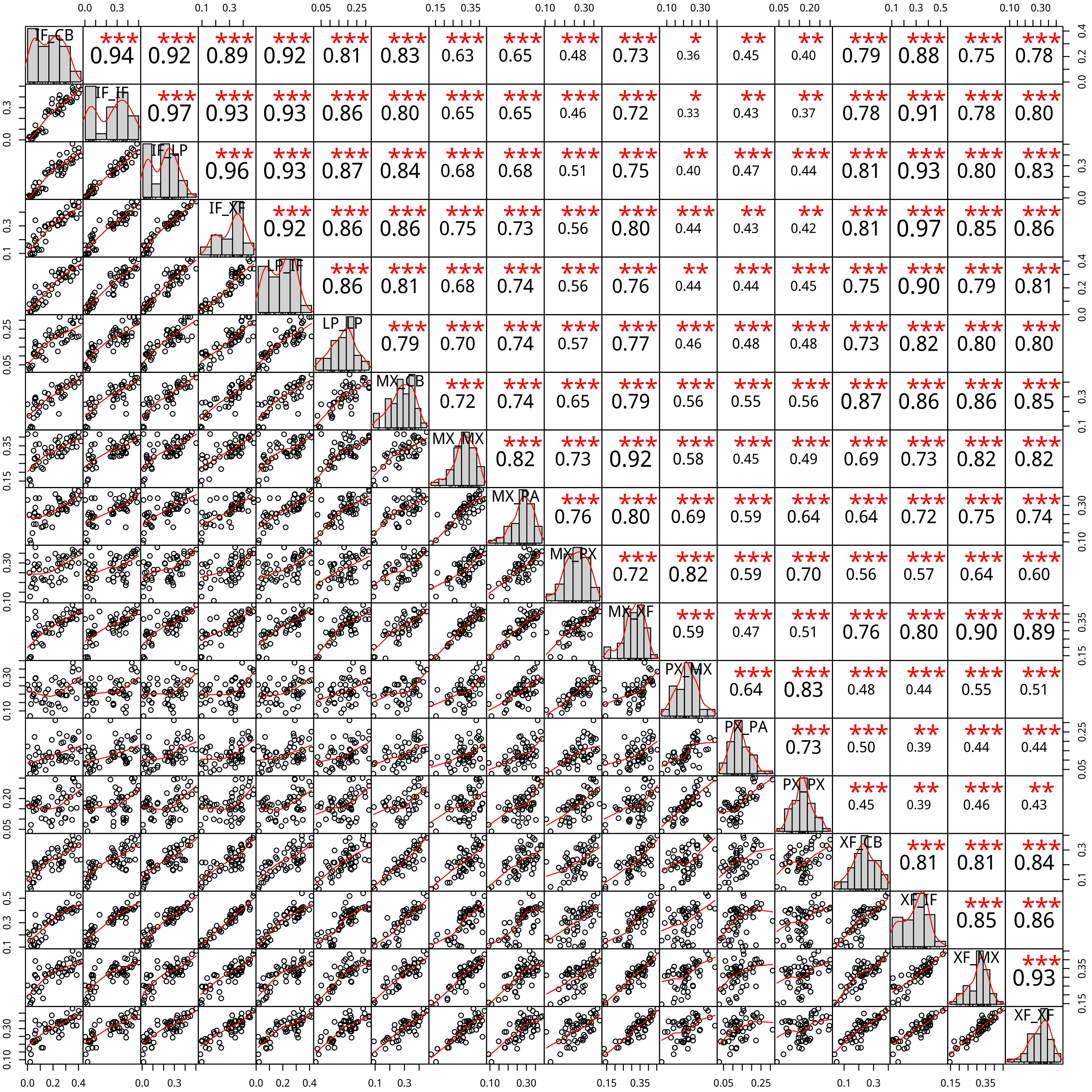

B

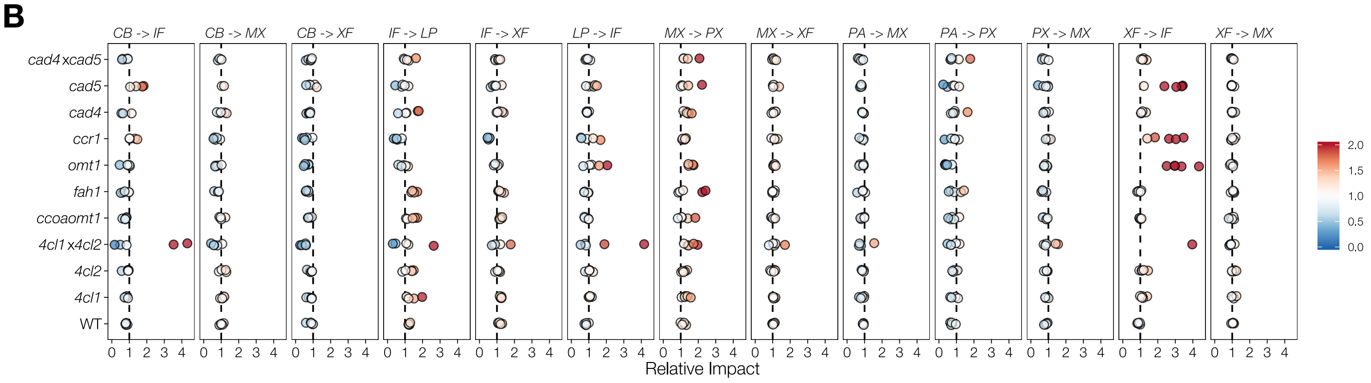

Figure S10 | Mathematical model matrix of cell-to-cell cooperation in *Arabidopsis*.

A Pearson Correlation matrix of all mathematically possible combinations of cell types in *A. thaliana*. The distribution of values for each specific cell wall in all the genotypes labelled “cell type”_“adjacent cell type” is shown on the diagonal. On the bottom of the diagonal the bivariate scatterplots are displayed each with a locally-weighted average (LOESS) fitted red line. Pearson’s *r* and *P*-value of the correlations are indicated above the diagonal (*** $P < 0.001$, ** $P < 0.01$, * $P < 0.05$, • $P < 0.1$).

B Relative impact of an adjacent cell type (arrow base) on the Wiesner test intensity in the cell walls of an adjacent cell type (arrowhead). CB (cambium), PA (pith parenchyma). There are 4 values (5.1, 10.1, 11.5, 52.4) outside the chosen scale in ccr1 (XF → IF).

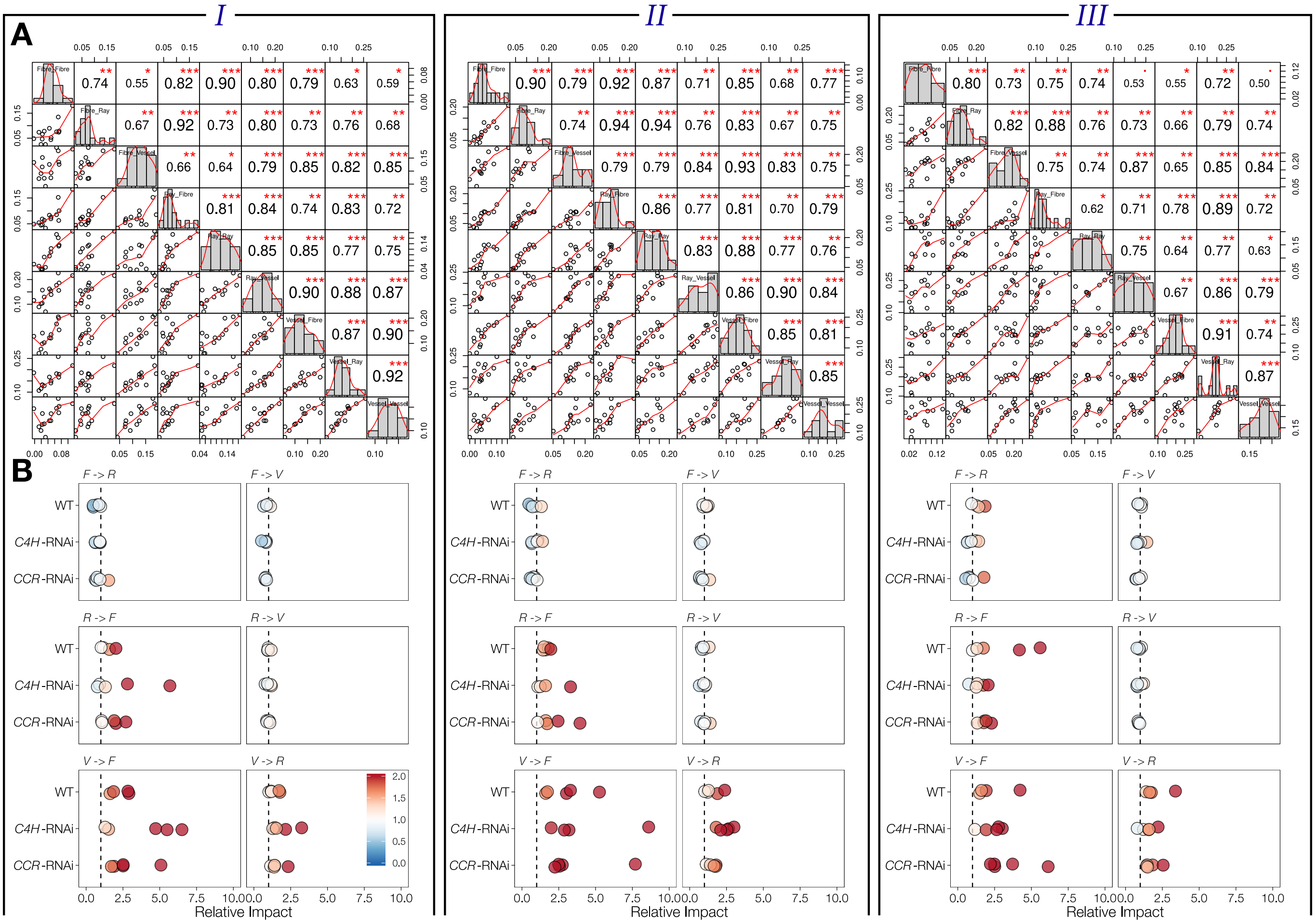

Figure S11 | Mathematical model matrix of cell-to-cell cooperation in poplar.

A Pearson Correlation matrix of all possible combinations of cell types in *P. tremuloides* × *P. tremula*, separated by developmental sector. The distribution of values for each specific cell wall in all the genotypes labelled "cell type"_"adjacent cell type" is shown on the diagonal. On the bottom of the diagonal the bivariate scatterplots are displayed each with a locally-weighted average (LOESS) fitted red line. Pearson's *r* and *P*-value of the correlations are indicated above the diagonal (*** $P < 0.001$, ** $P < 0.01$, * $P < 0.05$, • $P < 0.1$).

B Relative impact of an adjacent cell type (arrow base) on the Wiesner test intensity in the cell walls of an adjacent cell type (arrowhead). There is one value (21.9) outside the chosen scale in the WT (V → F, stage **III**).

Table S1 | Primers used for genotyping. PCR was done using classic Taq-polymerase master mix. PCR program: 94 °C (3 min) [94 °C (45 s), 57 °C (30 s), 72 °C (90 s)]×35, 72 °C (5 min).

Allele	Accession	Primer	Sequence
4cl1-1	SALK_142526	FW	AGCCTAAAATTCACACAACC
		RV	TCAGCATCACTCCTTTTGGT
4cl2-4	SALK_110197	FW	ATGACGACACAAGATGTGAT
		RV	CTAGTTCATTAATCCATTTG
ccr1-3	SALK_123-689	FW	TTGTGGAAATATTTCCGGTTG
		RV	GTGTCGTAGAGGCTTTGCTTG
cad4	SAIL_1265_A06	FW	TAGGTGAGGTGTTGGAAGT
		RV	ACATTCGTTGGACAAACAAGC
cad5	SAIL_776_B06	FW	GACCCCACATTGTTTGTTCAC
		RV	TCACCGCAATCAAATAAAACC
ccoaomt1	SALK_151507	FW	TTTTGTCGAACTTGGGAATTG
		RV	AATTTTCAAAGACCGGTGACC
fah1-2[a]	EMS mutant	FW	TGGTGTGTACATATATGGATGAAGAA
		RV	TAGCAAGAGTGGTGAATATGTGAAGT
omt1	SALK_135290	FW	TTGAAACTAGCTTGGTCGGTG
		RV	ATTCTTGATGGTGGGATTCC

[a] CAPS marker; genotyping by amplicon digestion with MseI

Table S2 | Multiple regression models of the Wiesner test dependency on the concentration of different lignin subunits.

Predictors	Adjusted R^2	BIC[a]
G	0.2734289	-16.41275
G + S	0.2447099	-14.88405
G + H	0.2052039	-14.32323
G + S + H	0.1452833	-12.59465

[a] Bayesian information criterion: lower values indicate a better model fit.
